# Infection risk and clinical outcomes with intravenous iron in adults with heart failure and iron deficiency: a systematic review and meta-analysis of randomised controlled trials

**DOI:** 10.1136/openhrt-2026-004486

**Published:** 2026-11-09

**Authors:** Saikat Mandal, Joyisa Deb, Vaidehi Prasanth, Aswin K Mohan, Justin Jose, So Ahng Yoon, Manideepa Maji, Asmita Goswami, Anindya Mukherjee, Arkadeep Dhali

**Affiliations:** 1 Translational Medical Sciences, University of Nottingham, Nottingham, UK; 2 NIHR Nottingham Biomedical Research Centre, Nottingham University Hospitals NHS Trust, Nottingham, UK; 3 Blood Centre, Apollo Excelcare Hospital, Guwahati, Assam, India; 4 Transfusion Medicine, All India Institute of Medical Sciences, New Delhi, Delhi, India; 5 Transfusion Medicine, All India Institute of Medical Sciences, Hyderabad, Telangana, India; 6 Cardiology, University Hospitals of Derby and Burton NHS Foundation Trust, Derby, UK; 7 School of Medicine, University of Nottingham, Nottingham, UK; 8 Hull York Medical School, University of Hull, Hull, UK; 9 Haematology, Hull University Teaching Hospitals NHS Trust, Hull, UK; 10 Cardiology, Leeds Teaching Hospitals NHS Trust, Leeds, UK; 11 Sheffield Teaching Hospitals NHS Foundation Trust, Sheffield, UK; 12 Imperial College London, London, UK

**Keywords:** HEART FAILURE, INFECTION, Meta-Analysis, Systematic Reviews as Topic, Pharmacology, Clinical

## Abstract

**Background:**

Intravenous iron may improve symptoms and reduce heart-failure hospitalisation, but its effect on infection risk remains uncertain. We evaluated infection risk and clinical outcomes in adults with heart failure and iron deficiency.

**Methods:**

We searched MEDLINE, Embase and Scopus from inception to 16 April 2026, with registry and regulatory searches through 18 October 2026. Randomised controlled trials comparing intravenous iron with placebo, usual care or oral iron were eligible. Participants experiencing infection and infection episodes were analysed separately using random-effects binomial-normal and Poisson-normal models, respectively. Common-effect models were prioritised for mortality and hospitalisation, with random-effects sensitivity analyses. Risk of bias and certainty were assessed using Risk of Bias 2 and Grading of Recommendations Assessment, Development and Evaluation.

**Results:**

Nineteen reports represented 18 independent cohorts. Three trials contributed participant-level data for any infection (1873 participants; OR 0.82, 95% CI 0.64 to 1.08; low certainty), and three contributed data for serious infection (1890 participants; OR 0.83, 95% CI 0.51 to 1.21; very low certainty). Infection-episode analyses were inconclusive: episode-burden ratios were 1.07 (95% CI 0.76 to 1.86; four trials) for any infection and 1.11 (0.82 to 1.42; seven trials) for serious infection, both with very low GRADE certainty. Intravenous iron probably reduced total first and recurrent heart-failure hospitalisations (three trials; rate ratio 0.78, 95% CI 0.67 to 0.90; moderate certainty). Intravenous iron probably has little or no effect on all-cause mortality (six trials; HR 0.93, 95% CI 0.84 to 1.03; moderate certainty). Quality-of-life findings generally favoured intravenous iron, but differences in instruments, score types and assessment times precluded pooling.

**Conclusions:**

Intravenous iron probably reduces heart-failure related hospitalisations. No clear increase in infection was observed. Incomplete reporting, inconsistent ascertainment and sparse data limit inference about infection safety. Future trials should prespecify standardised infection outcomes, and report affected participants, recurrent events and person-time.

**PROSPERO registration number:**

CRD420261355349.

**WHAT IS ALREADY KNOWN ON THIS TOPIC**

- Intravenous iron improves symptoms and reduces heart-failure hospitalisations, but inconsistent infection definitions and reporting have limited assessment of infection risk in this population.

**WHAT THIS STUDY ADDS**

- Intravenous iron was not associated with a clear increase in infection or serious infection, although the evidence was of low or very low certainty. It probably reduced heart-failure hospitalisations and may reduce cardiovascular hospitalisations.

**HOW THIS STUDY MIGHT AFFECT RESEARCH, PRACTICE OR POLICY**

- These findings support guideline-recommended intravenous iron treatment in eligible patients with heart failure and iron deficiency to reduce hospitalisations. The absence of a clear increase in infection provides some reassurance, although the limited certainty of the evidence warrants further assessment of infection risk in future clinical trials.

## Introduction

 Iron deficiency is one of the most prevalent and clinically important comorbidities in heart failure, affecting approximately half of patients with chronic heart failure and an even greater proportion of those admitted with acute decompensation.^1 2^ It may occur with or without anaemia and is associated with impaired exercise capacity, reduced health-related quality of life, recurrent hospitalisation and adverse prognosis.^1–3^ The pathophysiology is multifactorial, involving inadequate dietary intake, gastrointestinal congestion, impaired intestinal absorption, chronic inflammation, renal dysfunction and occult blood loss. Inflammatory activation disrupts hepcidin-ferroportin signalling, limiting iron absorption and mobilisation and contributing to functional iron deficiency despite preserved iron stores.^2 3^

Oral iron is inexpensive and widely available, but its effectiveness in heart failure may be limited by poor gastrointestinal absorption, inflammation, poor tolerability and suboptimal adherence. In the IRONOUT-HF randomised trial, high-dose oral iron produced only modest improvements in iron indices and did not improve exercise capacity in patients with heart failure with reduced ejection fraction.^4^ Intravenous iron bypasses these limitations and provides rapid iron repletion. Earlier trials of ferric carboxymaltose, including FAIR-HF and CONFIRM-HF, demonstrated improvements in symptoms, functional capacity and quality of life.^5 6^ Subsequent outcome trials, including AFFIRM-AHF, IRONMAN, HEART-FID and FAIR-HF2, have provided additional evidence on heart-failure hospitalisation and longer-term safety, although their effects on major cardiovascular endpoints have not been uniform.^7–10^ Accordingly, contemporary European Society of Cardiology guidelines recommend intravenous iron for symptomatic patients with heart failure with reduced or mildly reduced ejection fraction and iron deficiency to improve symptoms and quality of life, and support the use of ferric carboxymaltose or ferric derisomaltose to reduce heart-failure hospitalisation.^11^

Despite these established clinical benefits, concerns persist regarding the potential for intravenous iron to increase susceptibility to infection. Iron is essential for the replication and virulence of many bacterial and fungal pathogens, while host sequestration of circulating iron forms an important component of innate ‘nutritional immunity’.^12^ Administration of intravenous iron may transiently increase circulating non-transferrin-bound iron, promote oxidative stress and potentially impair selected neutrophil and macrophage functions. However, the relationship is biologically complex, as iron deficiency itself may impair immune responses. Newer iron-carbohydrate formulations are designed to release labile iron more slowly than older preparations. A recent review concluded that the clinical evidence remains inconclusive and that infection risk may vary according to iron formulation, administered dose, iron status and the underlying clinical condition.^13^

A systematic review of 154 randomised trials involving 32 762 participants found an increased risk of infection with intravenous iron across diverse clinical populations (risk ratio (RR) 1.16, 95% CI 1.03 to 1.29).^14^ Its applicability to heart failure is limited by differences in population, formulation, dose and infection ascertainment. A subsequent meta-analysis of six large heart-failure trials, with individual-participant data for five, reported an infection OR of 1.02 (95% CI 0.66 to 1.59).^15^ However, infection reports differ in severity threshold and in whether they enumerate affected participants or episodes. We therefore examined the heart-failure evidence by reporting unit and severity, using publications, trial registries and regulatory sources to document both usable and missing infection outcomes. Our primary objective was to estimate the risk of infection following intravenous iron in adults with heart failure and iron deficiency. Secondary outcomes included mortality, hospitalisation, health-related quality of life, length of hospital stay and anaphylaxis or serious hypersensitivity.

## Methods

### Study design, reporting and registration

This systematic review and meta-analysis was reported in accordance with the Preferred Reporting Items for Systematic Reviews and Meta-Analyses (PRISMA) 2020 statement^16^ and conducted in accordance with the Cochrane Handbook for Systematic Reviews of Interventions.^17^ The protocol was prospectively registered in PROSPERO on 30 March 2026 (CRD420261355349).

### Information sources and search strategy

MEDLINE and Embase were searched through Ovid, and Scopus was searched for records published from inception to 16 April 2026. The search combined controlled vocabulary and free-text terms for heart failure, iron deficiency or anaemia, intravenous iron, and individual preparations, including ferric carboxymaltose, ferric derisomaltose (iron isomaltoside), iron sucrose, sodium ferric gluconate and ferumoxytol. No infection-related outcome filter was applied because infection was often reported only in adverse-event, serious-adverse-event or supplementary safety tables. Complete database-specific strategies are provided in online supplemental appendix 1.

Reference lists of eligible reports and relevant reviews were examined. ClinicalTrials.gov and the WHO International Clinical Trials Registry Platform (ICTRP) were searched for additional details such as adverse event data and ongoing trials. Linked ClinicalTrials.gov and EU Clinical Trials Register results, public clinical study report synopses, regulatory assessments and companion publications were examined for infection data through 18 October 2026. Supplementary regulatory searches included the European Medicines Agency (EMA) and US Food and Drug Administration (FDA) websites, including Drugs@FDA. The search approach is provided in online supplemental appendix 1; documents contributing infection data and their extraction locations are listed in online supplemental table S6. Trial authors were contacted for additional data. Records were imported into Covidence (Veritas Health Innovation, Melbourne, Australia), and duplicates were removed before screening.

### Eligibility criteria

Eligible studies were randomised controlled trials enrolling adults with acute or chronic heart failure and iron deficiency, as defined by the investigators; evaluating an intravenous iron preparation and including a comparator receiving placebo, usual care, no iron or oral iron. No restriction was applied according to left ventricular ejection fraction, inpatient or outpatient setting, intravenous iron formulation, dosing schedule, cumulative dose or follow-up duration. Mixed-population trials were eligible only when data for participants with heart failure could be extracted separately.

Studies of oral iron without an intravenous-iron arm, single-arm or non-randomised studies, case reports, case series, reviews, editorials, dissertations, protocols without results and conference abstracts without sufficient arm-level data were excluded. Studies in which intravenous iron formed part of a combined intervention were excluded when its independent effect could not be isolated.

Eligibility was based on population, intervention, comparator and study design, with trials required to report at least one prespecified clinical or safety outcome. Trials without usable infection data were included and recorded as having no reported infection outcome; missing data were not treated as zero events. Multiple reports from the same trial were linked and treated as a single study, with companion publications and online supplemental materials used to complete outcome ascertainment.

### Study selection

Two reviewers independently screened each title and abstract and assessed all potentially eligible full-text reports against the prespecified criteria. Disagreements were resolved by discussion and, when required, consultation with a third reviewer. Reasons for full-text exclusion were recorded, and the selection process was summarised in a PRISMA 2020 flow diagram.

### Outcomes and infection classification

The primary outcome was the number of participants experiencing at least one infection of any severity during each trial’s reported safety follow-up. Participants with at least one serious infection were analysed separately. Broad infection categories, including the Medical Dictionary for Regulatory Activities (MedDRA) system organ class ‘Infections and infestations’, were distinguished from individual preferred terms. Total infection episodes and serious infection episodes were analysed separately to account for the different outcome units. Affected-participant counts were not summed across preferred terms, and episodes were not converted to participants. Analyses of specific serious-infection preferred terms and infection-related hospitalisation or death were exploratory.

Secondary clinical outcomes included total first and recurrent heart-failure hospitalisations; the number of participants with at least one heart-failure hospitalisation or readmission; all-cause mortality; all-cause hospitalisation; hospitalisation related to any cardiovascular cause; health-related quality of life assessed using validated instruments; length of hospital stay and anaphylaxis or serious hypersensitivity attributed to intravenous iron.

### Data extraction

Two reviewers independently extracted data using a piloted standardised form. Extracted variables included publication details, country, setting, trial design, sample size, participant characteristics, heart-failure phenotype and severity, left ventricular ejection fraction, definition of iron deficiency, baseline haemoglobin and iron indices, intravenous iron formulation, individual and cumulative doses, dosing schedule, comparator, follow-up and funding source.

For each outcome, reviewers recorded its definition, ascertainment, safety reporting window, affected participants or episodes, source-specific analysis denominator, person-time when available, and reported effect estimate with its 95% CI. The denominator attached to each safety outcome was retained rather than automatically substituting the randomised population. Counts reported only as rounded percentages were reconstructed when exactly one integer matched the stated denominator and precision; these records were flagged and excluded in sensitivity analyses. For FAIR-HF, counts of participants with serious infection were summed across mutually exclusive anaemia strata. Missing or unlisted infection terms were not imputed as zero. Alternative sources and unresolved discrepancies were retained in the extraction record (online supplemental table S6).

### Risk-of-bias and certainty assessments

Risk of bias was assessed using the revised Cochrane Risk of Bias 2 (RoB 2) tool,^18^ covering randomisation, deviations from intended interventions, missing outcome data, outcome measurement and selection of the reported result. Two reviewers independently assessed risk of bias, with disagreements resolved by discussion. Outcome-specific infection assessments distinguished bias within trial results from incomplete reporting across the evidence base. Domain judgements, source-specific rationales and recorded signalling-question responses are provided in online supplemental tables S2, S3 and S11. Reconstruction of an unambiguous integer count from a rounded percentage was treated as an extraction issue and examined in sensitivity analysis, rather than as evidence of selective reporting.

Certainty was assessed using the Grading of Recommendations Assessment, Development and Evaluation (GRADE) approach.^19^ Randomised evidence started at high certainty and was downgraded, where appropriate, for risk of bias, inconsistency, indirectness, imprecision or missing results across studies. Reasons were recorded separately for each domain (online supplemental table S4).

### Effect measures and statistical analysis

Meta-analysis was undertaken whenever at least two clinically and methodologically comparable trials reported the same outcome. Participant-level infection was defined as the number of participants experiencing at least one infection and was analysed using a binomial likelihood. Other participant-level dichotomous outcomes were summarised as RRs with 95% CIs. Total infection-event counts, for which a participant could contribute more than once, and trial-reported recurrent-event rate ratios were analysed separately from participant-level outcomes. ORs, RRs, event-count ratios and recurrent-event rate ratios were not combined within the same model.

Arm-specific person-time and the distribution of recurrent infections within participants were unavailable for the broad infection-episode datasets. Episode models therefore used the source-specific number of participants as the exposure denominator and estimated episode burden per participant over each trial’s reporting window. Absolute mortality effects were estimated at 12 months using control risks from CONFIRM-HF and AFFIRM-AHF. Corresponding risks with intravenous iron were calculated as 1−(1−baseline risk)^HR^, assuming proportional hazards. Absolute risk differences were calculated by subtracting the baseline risk from the estimated intervention risk. For other binary outcomes, absolute effects were derived from the pooled control risk and the corresponding pooled relative effect. These estimates are approximate because observation periods differed between trials.

#### Primary participant-level infection analysis

Any-infection and serious-infection participant counts were analysed separately using a binomial-normal generalised linear mixed model (GLMM) with fixed study intercepts, a normally distributed random treatment effect and logit link. Treatment was coded symmetrically as +½ and −½. Models were fitted by maximum likelihood using 60-point Gauss–Hermite quadrature. The exponentiated treatment coefficient estimates an OR. CIs were obtained by profiling the likelihood while re-estimating heterogeneity; two-sided p values were obtained from likelihood-ratio tests. Zero-event arms were retained without continuity correction, and double-zero studies were described separately.

### Sensitivity and influence analyses

Sensitivity analyses for participant-level infection used log-RR random-effects meta-analysis with restricted maximum likelihood (REML) and modified Hartung–Knapp inference, conventional REML–Wald inference and Mantel–Haenszel common-effect pooling.^20 21^ REML estimates between-study variance without relying on a common-effect assumption. Modified Hartung–Knapp inference allows for uncertainty in this estimate; its variance factor was bounded below at one, with t inference using k−1 degrees of freedom. With few trials, these intervals may be very wide and were interpreted alongside profile-likelihood GLMM and conventional Wald intervals. The binomial random-effects GLMM provided the principal infection estimate.

Leave-one-out analyses omitted each trial in turn from syntheses containing at least three trials. Additional sensitivity analyses excluded reconstructed CONFIRM-HF counts; conditionally included FAIR-HF2 participant counts from the published meta-analysis^15^; restricted any-infection participants to nominal 52-week studies; excluded IRONMAN from serious-infection participants; restricted episode analyses by reporting window or source and excluded FAIR-HFpEF. Analyses restricted to results at low risk of bias excluded those judged to raise some concerns. Formulation, recruitment setting, blinding, comparator and reporting window were mapped for each infection synthesis. Stratified estimates were descriptive: sparse strata and confounding between characteristics precluded reliable interaction tests or meta-regression (online supplemental table S9).

### Infection episodes and clinical outcomes

All principal infection analyses used random-effects models because definitions, severity thresholds, ascertainment and reporting windows varied. Any-severity and serious infection episodes were pooled separately using Poisson-normal GLMMs with fixed study intercepts, a random treatment effect and log(number of participants) as the offset.^22 23^ Between-study variance was estimated by maximum likelihood; profile-likelihood CIs and likelihood-ratio tests were used. Sensitivity analyses used Mantel–Haenszel pooling and variance inflation under assumed negative-binomial dispersion.

Common-effect models provided the principal estimates for mortality and hospitalisation under an assumed shared relative treatment effect within each synthesis. This choice reflected the assumption of a shared relative effect on comparable mortality and admission endpoints after iron repletion; it was independent of heterogeneity tests. Population, formulation, comparator and follow-up differences were considered when interpreting results. Binary clinical outcomes used Mantel–Haenszel RRs; mortality HRs and recurrent heart-failure hospitalisation rate ratios used generic inverse variance on the log scale. Random-effects sensitivity analyses used REML with modified Hartung–Knapp inference.

Four mortality HRs were extracted from original trial reports; the FAIR-HF and AFFIRM-AHF HRs were obtained from the 2025 meta-analysis.^15^ A source-restricted analysis included only original-report HRs. Recurrent heart-failure hospitalisation rate ratios were pooled separately at 12 months and over complete follow-up. Binary mortality and hospitalisation analyses were repeated using outcomes observed for at least 24 weeks. This restriction excluded short observation windows but did not standardise follow-up or account for censoring (online supplemental tables S7 and S8).

### Zero-event arms, heterogeneity and analysis hierarchy

No continuity correction was used in the binomial or Poisson GLMMs or Mantel–Haenszel pooled estimates. For inverse-variance log-RR analyses, 0.5 was added to all four cells of a trial with a zero-event arm. Double-zero trials were described separately because they do not estimate a relative effect. Heterogeneity was summarised using Cochran’s Q, I² and model-specific τ².^24^ Throughout, Q and I² for binary and episode counts used inverse-variance log effects from trials without a zero-event arm; HR and rate-ratio analyses used the reported log effects. Prediction intervals were not reported for sparse infection syntheses because between-study variance was too poorly estimated to support a useful prediction.

The principal any-infection analysis included three trials with compatible participant counts; serious-infection participants were evaluated in a separate three-trial analysis. FAIR-HF2 participant counts from a secondary individual-participant-data (IPD) source were included in a sensitivity analysis because detailed severity and ascertainment definitions could not be independently confirmed. Four trials contributed any-severity infection episodes and seven contributed serious infection episodes; episode and preferred-term analyses were exploratory. Non-serious registry episodes were reported descriptively where reporting thresholds or unresolved numerical inconsistencies limited pooling. EFFECT-HF serious and non-serious episodes were not combined because overlap between the reporting sections was unresolved.

For the seven trials reporting arm-wise means and SD, health-related quality-of-life outcomes were presented as individual standardised mean differences using Hedges’ g in an unpooled forest plot, with one estimate selected per trial. Scores from instruments in which lower values indicated better health were reversed so that positive estimates consistently favoured intravenous iron. No pooled estimate was presented because the instruments measured differing constructs, final and change-from-baseline scores would have been combined, assessment ranged from 2 weeks to 20 months, and statistical heterogeneity was substantial (I²=82%). Arm-wise standardised mean differences were compared with the trials’ baseline-adjusted treatment effects when reported. Adjusted model estimates, medians with IQRs, and graphically or narratively reported results were retained descriptively but not converted.

### Publication bias assessment

Funnel plots and Egger’s regression were used only for binary outcomes reported by at least 10 trials.^17^ Trial-level log RRs were plotted against their standard errors, with a 0.5 continuity correction for zero-event arms. These exploratory assessments have limited power and cannot exclude bias from missing results. Infection outcomes were not assessed with funnel-asymmetry tests because too few trials contributed.

### Software and reproducibility

Analyses were performed in R V.4.3.3 using meta V.8.0.1, metafor V.4.4.0 and lme4 V.1.1.35.1.^25–27^ Infection models and additional clinical analyses were implemented in Python 3 using NumPy and SciPy. Standardised mean differences were calculated as Hedges’ g. Model specifications, data sources and numerical results are provided in online supplemental methods and tables S5–S11. Additional analyses were post hoc, as specified in online supplemental methods. P values were two-sided; no adjustment was made for multiple comparisons.

## Results

### Study selection

The searches identified 4166 records: 2164 from Embase, 1212 from MEDLINE and 790 from Scopus. Covidence identified 1392 duplicate records, and 10 additional duplicates were removed manually. Of the 2764 records screened by title and abstract, 2495 were excluded. All 269 reports sought for retrieval were obtained and assessed for eligibility; 250 were excluded because of wrong study design (n=133), wrong outcomes (n=87), wrong patient population (n=10), wrong intervention (n=8), wrong comparator (n=5), duplicate report (n=4), wrong indication (n=2) or wrong route of administration (n=1). Nineteen trial reports were included (figure 1); their principal characteristics are summarised in table 1 and detailed in online supplemental table S1.^5–10 28–40^ These 19 reports represent 18 independent cohorts, as Toblli *et al*^28^ reports a subset of the participants in the expanded Toblli *et al*^31^ cohort; the two reports never contribute to the same analysis.

**Figure 1:**
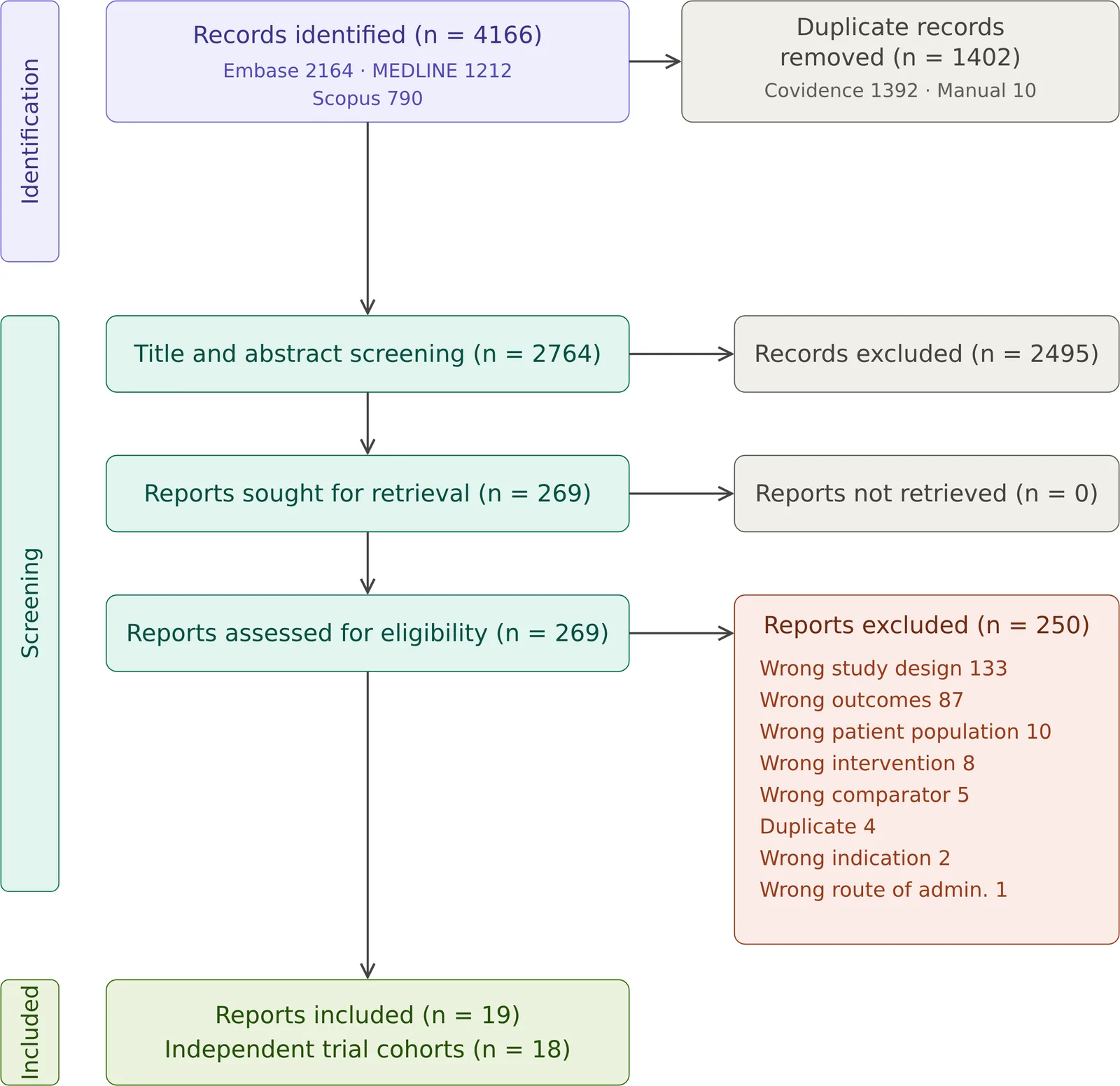
Preferred Reporting Items for Systematic Reviews and Meta-Analyses (PRISMA) 2020 flow diagram for the identification, screening and inclusion of randomised controlled trials of intravenous iron in heart failure.

**Table 1:** Characteristics of the included studies

Study/report	Population and design	N	Intervention/comparator	Follow-up
Toblli et al*^28^	Chronic HFrEF, CKD and iron-deficiency anaemia; double-blind	40	Iron sucrose/saline placebo	6 months
FERRIC-HF^29^	Ambulatory symptomatic HFrEF; open-label	35	Iron sucrose/no intravenous iron	16 weeks
FAIR-HF^5^	Ambulatory NYHA II–III HFrEF; double-blind	459	Ferric carboxymaltose/saline placebo	24 week treatment; safety to 26 weeks
IRON-HF^30^	Chronic HFrEF with iron-deficiency anaemia; three-arm RCT	23†	Iron sucrose/placebo	3 months
CONFIRM-HF^6^	Ambulatory symptomatic HFrEF; double-blind	304	Ferric carboxymaltose/saline placebo	52 weeks
Toblli et al*^31^	Chronic HFrEF, CKD and iron-deficiency anaemia; double-blind	60	Iron sucrose/saline placebo	6 months
EFFECT-HF^32^	Ambulatory HFrEF; open-label, blinded endpoint	172‡	Ferric carboxymaltose/standard care	24 weeks
Youssef *et al*^33^	Acute decompensated HFrEF; placebo-controlled	60	Iron sucrose/saline placebo	3 months
PRACTICE-ASIA-HF^34^	Stabilised after acute HF admission; double-blind pilot	50	Ferric carboxymaltose/saline placebo	12 weeks
FERRIC-HF II	Symptomatic chronic HFrEF; double-blind mechanistic RCT	40	Ferric derisomaltose/saline placebo	2 weeks
FCM-HF-IN	Symptomatic chronic HFrEF; open-label	70	Ferric carboxymaltose/standard care	12 weeks
AFFIRM-AHF^7^	Stabilised after acute HF; double-blind	1132	Ferric carboxymaltose/placebo	52 weeks
IRON-CRT^37^	HFrEF after cardiac resynchronisation therapy; double-blind	75	Ferric carboxymaltose/placebo	3 months
SFGC pilot	Hospitalised acute HF; single-blind pilot	34	Sodium ferric gluconate/standard care	HF readmission at 6 months
IRONMAN^8^	HFrEF; mixed outpatient and hospital recruitment; open-label, blinded endpoint	1137	Ferric derisomaltose/usual care	Median 2.7 years
HEART-FID^9^	Ambulatory HFrEF; double-blind	3065	Ferric carboxymaltose/placebo	Median 1.9 years
FAIR-HFpEF^39^	Symptomatic HFpEF; double-blind pilot	39	Ferric carboxymaltose/saline placebo	24-week efficacy; safety to 52 weeks
FAIR-HF2	Stable chronic HFrEF; double-blind	1105	Ferric carboxymaltose/saline placebo	Median 16.6 months
Sung *et al*^40^	Acute decompensated HFrEF; open-label	65	Iron sucrose/standard care	4 weeks; events to 3 months

* Toblli *et al* is a subset of Toblli *et al*; the reports never contributed to the same synthesis.

† The review comparison used the intravenous-iron (n=10) and placebo (n=6) arms.

‡ EFFECT-HF enrolled 174 participants; 172 entered the principal analysis. Mortality and clinical counts use 88/86 from the original outcome table; registry safety counts use another source population.

CKD, chronic kidney disease; HF, heart failure; HFpEF, heart failure with preserved ejection fraction; HFrEF, heart failure with reduced ejection fraction; RCT, randomised controlled trial.

### Availability of infection outcomes

Eight of the 18 independent cohorts contributed to at least one pooled infection analysis. Three reported compatible counts of participants with any infection, and three reported participants with serious infection; four reported episodes of any severity and seven reported serious episodes. FAIR-HF2 participant counts were included only in a conditional sensitivity analysis. Using source-reported cohort sizes, the eight contributing cohorts represented 7413 of 7925 participants (94%), but the primary any-infection analysis represented trials enrolling 1895 participants (24%); its source-specific safety population comprised 1873 participants. The 10 non-contributing cohorts were smaller (median 55 participants) and shorter (median 13 weeks), and 6 used iron sucrose or sodium ferric gluconate. The limited availability of the primary outcome remained a potential source of bias (online supplemental figure S4 and tables S6 and S10).

### Infection in participants

#### Any infection

FAIR-HF, AFFIRM-AHF and CONFIRM-HF contributed 1873 participants. At least one infection occurred in 192 of 1016 intravenous-iron recipients and 196 of 857 controls (OR 0.82, 95% CI 0.64 to 1.08; p=0.107; I²=0%; figure 2A). The evidence was of low certainty because of imprecision and missing results across studies. CONFIRM-HF counts of 40/152 versus 51/152 were reconstructed from reported percentages.

**Figure 2:**
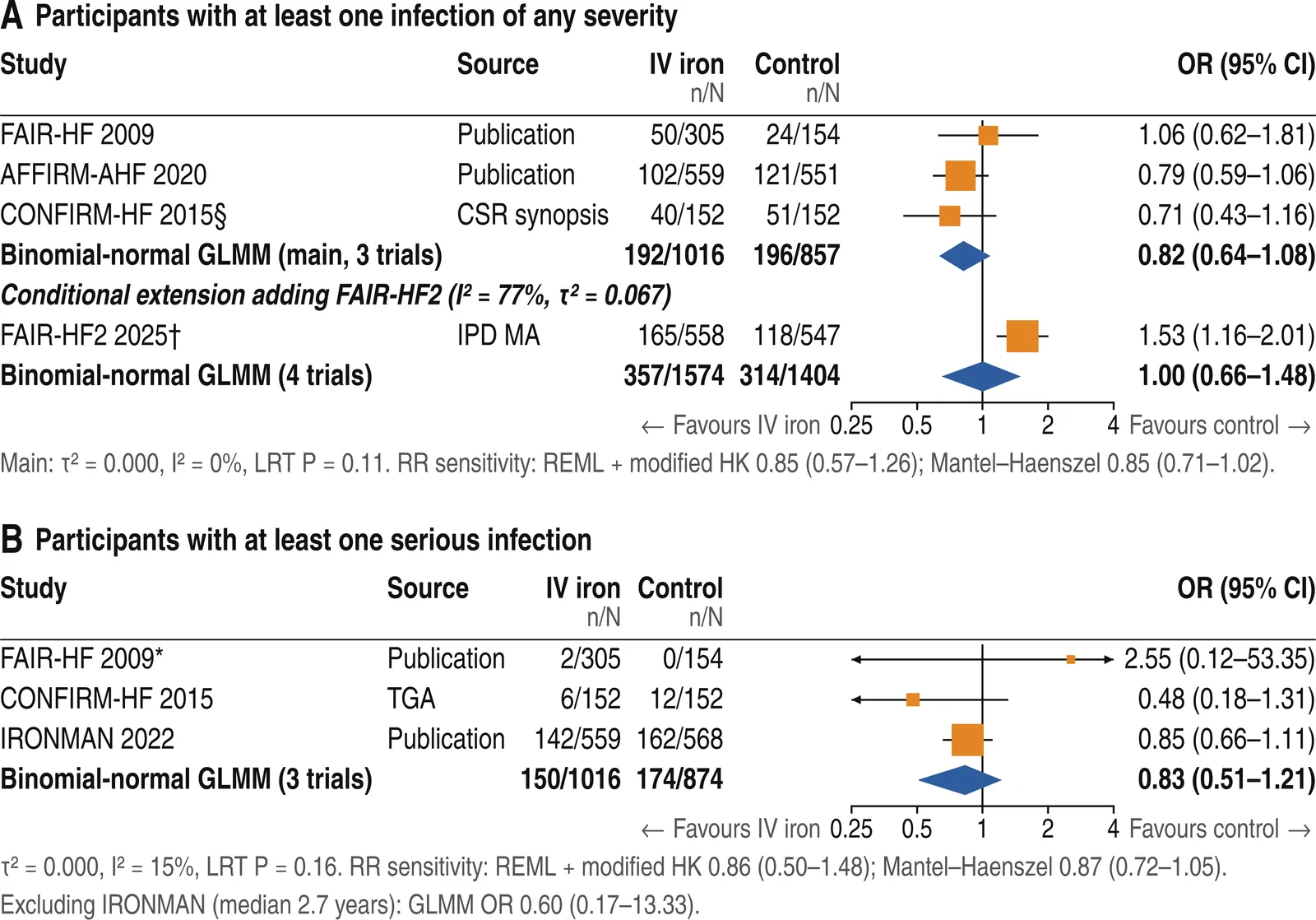
Infection outcomes in participants. (A) Participants with at least one infection of any severity in three trials, with a sensitivity analysis adding FAIR-HF2. (B) Participants with at least one serious infection. Blue diamonds show binomial-normal GLMM ORs with profile-likelihood 95% CIs. RR sensitivity estimates are shown beneath each panel. Square areas reflect inverse-variance weights for the displayed trial estimates, rather than GLMM weights. *A 0.5 correction was used to display the zero-cell trial; the pooled GLMM was uncorrected. †Counts from a published IPD meta-analysis.^15^ §Counts uniquely reconstructed from rounded percentages in the CSR synopsis. CSR, clinical study report; GLMM, generalised linear mixed model; HK, Hartung–Knapp; IPD MA, individual-participant-data meta-analysis; LRT, likelihood-ratio test; REML, restricted maximum likelihood; RR, risk ratio; TGA, Therapeutic Goods Administration.

In sensitivity analyses, the REML-modified Hartung–Knapp RR was 0.85 (95% CI 0.57 to 1.26), and the Mantel–Haenszel common-effect RR was 0.85 (0.71–1.02). Excluding reconstructed CONFIRM-HF counts gave an OR of 0.85 (0.62–1.29). Restricting the analysis to AFFIRM-AHF and CONFIRM-HF, both nominal 52-week studies, gave an OR of 0.77 (0.55–1.03). Leave-one-out ORs ranged from 0.77 to 0.86 (online supplemental figure S7A and table S5).

Conditional inclusion of FAIR-HF2 participant counts from the published meta-analysis^15^ (165/558 vs 118/547) gave an OR of 1.00 (95% CI 0.66 to 1.48; I²=77%; figure 2A). The infection definition, analysis population and reporting window could not be independently matched to those of the primary analysis. The discrepancy between these participant counts and the primary report’s serious-episode totals does not establish that the counts represent any-severity infection. Four-trial influence analyses are shown in online supplemental figure S7B.

All three primary any-infection trials used ferric carboxymaltose, placebo control and double blinding; AFFIRM-AHF alone recruited after acute decompensation. In the serious-infection participant analysis, IRONMAN introduced ferric derisomaltose, usual care, open-label treatment, mixed outpatient and hospital recruitment and longer follow-up. Formulation effects therefore could not be separated from these design differences. Stratified estimates are descriptive; subgroup-difference tests were not performed (online supplemental table S9 and figure S11).

#### Serious infection

FAIR-HF, CONFIRM-HF and IRONMAN contributed 1890 participants. Serious infection affected 150 of 1016 intravenous-iron recipients and 174 of 874 controls (OR 0.83, 95% CI 0.51 to 1.21; p=0.162; I²=15%; figure 2B). The evidence was very uncertain. Sensitivity estimates were RR 0.86 (95% CI 0.50 to 1.48) with REML-modified Hartung–Knapp and RR 0.87 (0.72–1.05) with Mantel–Haenszel pooling. Excluding IRONMAN, whose result was judged to raise some concerns, gave an OR of 0.60 (0.17–13.33). Leave-one-out ORs ranged from 0.60 to 0.86 (online supplemental figure S7C and table S5).

#### Infection-related hospitalisation and death

In CONFIRM-HF, infection adverse events leading to hospitalisation affected 5/152 intravenous-iron recipients and 12/152 controls (RR 0.42, 95% CI 0.15 to 1.15); counts were reconstructed from the reported percentages. IRONMAN reported a HR of 0.76 (95% CI 0.59 to 0.98) for first hospitalisation with infection as the main cause.^41^ Infection-related death and recurrent-hospitalisation estimates from IRONMAN are shown in online supplemental figure S8C.

### Infection episodes and specific serious infections

Four trials reported 491 infection episodes of any severity with intravenous iron and 406 with control (episode-burden ratio 1.07, 95% CI 0.76 to 1.86; I²=82%). Seven reported 440 and 380 serious episodes, respectively (episode-burden ratio 1.11, 95% CI 0.82 to 1.42; I²=49%; online supplemental figure S8A,B). Both estimates were of very low certainty. The Mantel–Haenszel serious-episode ratio was 1.15 (95% CI 1.00 to 1.32; p=0.048); assumed negative-binomial dispersion of θ=0.5 gave 1.14 (0.98–1.33).

In trials with confirmed reporting windows of ≤52 weeks, the serious-episode ratio was 0.81 (95% CI 0.57 to 1.41), compared with 1.24 (1.03 to 1.49) in two trials with longer windows. Both longer-window trials enrolled chronic heart failure, so follow-up and setting could not be separated. FAIR-HFpEF registry data were omitted from window strata because the reporting horizon was unconfirmed. Restriction to publication or regulatory sources gave 1.16 (0.55–2.01). Excluding FAIR-HFpEF gave 1.04 (0.70–1.47) for any episodes and 1.11 (0.80–1.41) for serious episodes. Restriction to results judged at low risk of bias gave a serious-episode ratio of 1.09 (0.75–1.39) and a serious-pneumonia OR of 1.26 (0.53–2.29). Additional influence analyses are provided in online supplemental figure S7 and table S5.

For specific serious infections, pooled ORs were 1.34 (95% CI 0.69 to 2.25) for pneumonia, 1.21 (0.37–2.36) for sepsis, 1.31 (0.20–2.77) for urinary tract infection, 0.66 (0.13–2.94) for bacteraemia and 1.79 (0.41–7.46) for septic shock (online supplemental figure S6).

### Secondary clinical outcomes

#### All-cause mortality

Six trials involving 7175 participants provided all-cause mortality HRs. The principal common-effect estimate was 0.93 (95% CI 0.84 to 1.03; p=0.14; I²=0%), compared with 0.93 (0.81–1.06) using REML-modified Hartung–Knapp (figure 3A). Intravenous iron probably has little or no effect on mortality, although a modest benefit remains possible (moderate certainty). Restriction to the four HRs reported in the original trial publications gave 0.92 (0.83–1.03); leave-one-out estimates ranged from 0.92 to 0.95 (online supplemental figure S10A). The binary analysis included 14 trials and 7684 participants (RR 0.95, 95% CI 0.88 to 1.04; random-effects RR 0.96, 0.87–1.05). Restriction to eight trials with at least 24 weeks of follow-up (7383 participants) gave an RR of 0.95 (0.87–1.04; random-effects RR 0.95, 0.86–1.06; online supplemental figure S9A). At illustrative 12-month risks of 93 and 175 deaths per 1000, the pooled HR corresponds to 6 fewer deaths per 1000 (14 fewer to 2 more) and 12 fewer (26 fewer to 4 more), respectively (table 2).

**Figure 3:**
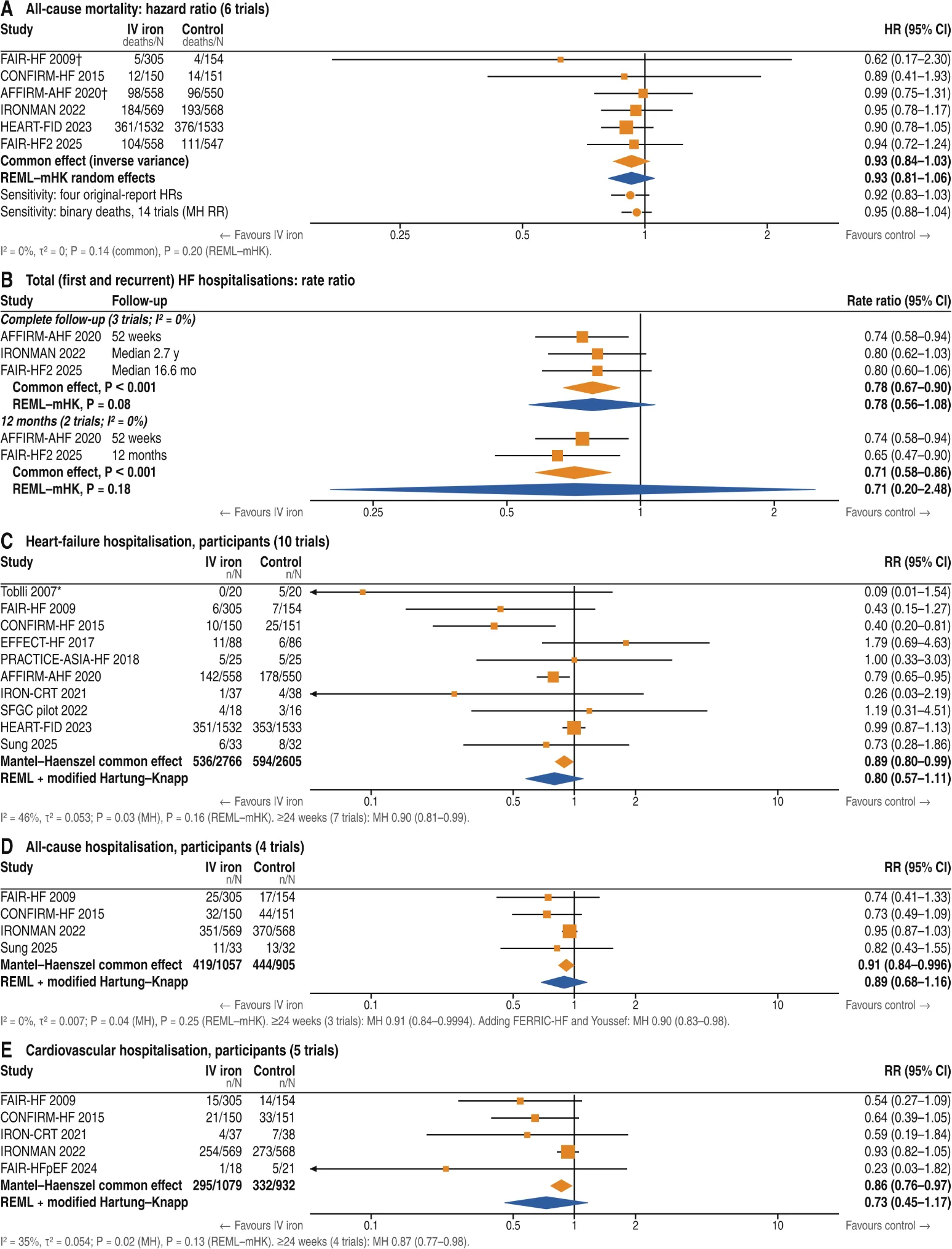
Clinical outcomes. (A) All-cause mortality. (B) Total first and recurrent heart-failure hospitalisations over complete follow-up and at 12 months. (C) Participants with heart-failure hospitalisation. (D) Participants with all-cause hospitalisation. (E) Participants with cardiovascular hospitalisation. Orange diamonds show principal common-effect estimates; blue diamonds show REML–mHK sensitivity estimates. Square areas reflect inverse-variance weights in A and Mantel–Haenszel weights in C–E. †Mortality HR from the 2025 meta-analysis.^15^ *Zero-event arm displayed with a 0.5 correction; pooled Mantel–Haenszel estimates are uncorrected. I^2^ excludes zero-event arms in C–E. HF, heart failure; mHK, modified Hartung–Knapp; REML, restricted maximum likelihood; RR, risk ratio.

**Table 2:** Summary of findings

Outcome	Trials; N	Principal effect (95% CI)	Control risk and absolute effect per 1000 (95% CI)	Certainty	Interpretation
Any infection in participants	3; 1873	OR 0.82 (0.64 to 1.08)	229/1000; 34 fewer (70 fewer to 13 more)	Low	May have little or no effect; incomplete reporting limits inference.
Serious infection in participants	3; 1890	OR 0.83 (0.51 to 1.21)	199/1000; 28 fewer (87 fewer to 32 more)	Very low	The effect is very uncertain.
All-cause mortality	6; 7175	HR 0.93 (0.84 to 1.03)	At 12 months: baseline 93/1000, 6 fewer (14 fewer to 2 more); baseline 175/1000, 12 fewer (26 fewer to 4 more)	Moderate	Probably has little or no effect; modest benefit remains possible.
Total first and recurrent HF hospitalisations	3; 3350	Rate ratio 0.78 (0.67 to 0.90)	Not estimated without a common baseline event rate	Moderate	Probably reduces hospitalisations; random-effects CI includes no effect.
At least one HF hospitalisation	10; 5371	RR 0.89 (0.80 to 0.99)	228/1000; 25 fewer (45 to 3 fewer)	Moderate	Probably reduces hospitalisation; random-effects CI includes no effect.
All-cause hospitalisation	4; 1962	RR 0.91 (0.84 to 0.996)	491/1000; 43 fewer (81 to 2 fewer)	Low	May reduce hospitalisation; effect is imprecise.
Cardiovascular hospitalisation	5; 2011	RR 0.86 (0.76 to 0.97)	356/1000; 49 fewer (84 to 10 fewer)	Low	May reduce hospitalisation; effect is imprecise.

Principal estimates are random-effects binomial-normal GLMM ORs for infection and common-effect HRs, RRs or rate ratios for clinical outcomes. Random-effects models for clinical outcomes provide sensitivity estimates. Binary absolute effects use the crude pooled control risk over trial-specific windows and are approximate. Mortality effects use the pooled HR and two stated 12-month baseline risks, assuming proportional hazards; they are illustrative estimates rather than directly observed pooled risk differences. Some hospitalisation CI limits are shown with additional decimals to avoid rounding an upper limit below one to 1.00. Domain judgements are provided in online supplemental table S4.

GLMM, generalised linear mixed model; HF, heart failure; RCT, randomised controlled trial; RR, risk ratio.

#### Heart-failure hospitalisation outcomes

Three trials involving 3350 participants reported total first and recurrent heart-failure hospitalisations over complete follow-up. Intravenous iron probably reduced hospitalisations (rate ratio 0.78, 95% CI 0.67 to 0.90; I²=0%; moderate certainty). At 12 months, AFFIRM-AHF and FAIR-HF2 gave a common-effect rate ratio of 0.71 (95% CI 0.58 to 0.86; I²=0%) (figure 3B).

Ten trials involving 5371 participants reported at least one heart-failure hospitalisation or readmission (536/2766 vs 594/2605). The common-effect RR was 0.89 (95% CI 0.80 to 0.99; I²=46.4%; moderate certainty, figure 3C). Restriction to seven trials with at least 24 weeks of follow-up (5181 participants) gave an RR of 0.90 (0.81–0.99; online supplemental figure S9B).

#### All-cause hospitalisation

Four trials involving 1962 participants reported compatible all-cause hospitalisation counts (419/1057 vs 444/905). Intravenous iron may reduce all-cause hospitalisation (RR 0.91, 95% CI 0.84 to 0.996; p=0.040; I²=0%; low certainty), but the random-effects estimate was imprecise (0.89, 0.68–1.16; figure 3D). Toblli *et al*^31^ was excluded because the intravenous-iron numerator was unreported; FERRIC-HF reported events rather than clearly unique participants, and the admission cause in Youssef *et al*^33^ was unspecified. Adding the latter two records gave an RR of 0.90 (0.83–0.98; online supplemental figure S10B). Restriction to three trials with at least 24 weeks of follow-up (1897 participants) gave an RR of 0.91 (0.84–0.9994; online supplemental figure S9C).

#### Hospitalisation related to any cardiovascular cause

Five trials involving 2011 participants reported cardiovascular hospitalisation (295/1079 vs 332/932). Intravenous iron may reduce cardiovascular hospitalisation (RR 0.86, 95% CI 0.76 to 0.97; I²=35.4%; low certainty; figure 3E). Restriction to four trials with at least 24 weeks of follow-up (1936 participants) gave an RR of 0.87 (0.77–0.98; online supplemental figure S9D).

### Risk of bias

In the study-level assessment, three reports were judged at low overall risk of bias, and 16 raised some concerns; none was rated high risk (online supplemental figure S1 and table S2). Risk of bias varied by infection outcome and data source. All three primary any-infection results were rated low risk. IRONMAN serious-infection participants and EFFECT-HF registry outcomes raised measurement concerns because of open-label ascertainment. FAIR-HFpEF registry outcomes raised concerns about missing data and differences between published and registry populations. FAIR-HF2’s conditional participant-level result raised concerns because its definition and window could not be confirmed. Domain rationales and recorded signalling responses are provided in online supplemental tables S3 and S11.

### Outcomes not quantitatively synthesised

#### Health-related quality of life

Health-related quality of life was reported using heterogeneous instruments: the Kansas City Cardiomyopathy Questionnaire (KCCQ), Minnesota Living with Heart Failure Questionnaire (MLHFQ) and EuroQol measures at time points ranging from 2 weeks to 20 months; patient global assessment and NYHA class were not treated as quality-of-life instruments. Toblli 2007, FAIR-HF, CONFIRM-HF, FCM-HF-IN, AFFIRM-AHF, IRON-CRT, FAIR-HF2 and Sung *et al* reported improvement in at least one measure with intravenous iron. IRONMAN reported better MLHFQ overall and physical scores at 4 months (adjusted differences −3.33, 95% CI −6.67 to 0.00, and −1.98, −3.42 to −0.54) but no difference at 20 months or in EQ-5D at either time point, while PRACTICE-ASIA-HF, FAIR-HFpEF, FERRIC-HF and FERRIC-HF II showed no clear between-group difference. HEART-FID reported no validated instrument.

Seven trials reported means and SD in both arms (online supplemental figure S5). Standardised mean differences ranged from Hedges’ g 0.22 (95% CI 0.10 to 0.33) in FAIR-HF2 to 2.35 (1.53–3.16) in Toblli 2007. Estimates were not pooled because instruments, score types and assessment times differed (I²=82%). Excluding Toblli 2007 reduced I² to 40% (Cochran’s Q p=0.14), with estimates in the remaining six trials ranging from 0.22 to 0.87. In FAIR-HFpEF, the unadjusted change-score estimate was g=0.87, whereas the trial’s adjusted between-group difference was 6.5 KCCQ points (95% CI −3.8 to 16.7). Only Toblli 2007 contributed quality-of-life data from the overlapping Toblli cohorts.

#### Length of hospital stay

None of the 18 independent trial cohorts reported an extractable arm-level comparison of length of hospital stay that met the review definition. This outcome was therefore not meta-analysed.

#### Anaphylaxis and serious hypersensitivity

Available comparative data contained no anaphylaxis or serious hypersensitivity events, or lacked comparator counts, precluding a pooled relative effect. FERRIC-HF and FERRIC-HF II reported no anaphylactic reactions in either arm (0/24 vs 0/11 and 0/21 vs 0/19, respectively). FAIR-HF, CONFIRM-HF, FCM-HF-IN, PRACTICE-ASIA-HF and FAIR-HFpEF reported no severe allergic or hypersensitivity reactions attributed to treatment in either arm. HEART-FID reported five hypersensitivity events among 1532 ferric carboxymaltose recipients: three were judged probably treatment-related, one was severe but not serious, and two were angioedema events. Comparator counts for these terms were not reported. IRONMAN reported one infusion reaction that was not classified as anaphylaxis or serious hypersensitivity.

### Summary of findings

Principal effects, illustrative absolute effects and certainty ratings are presented in table 2. Certainty was low for participants with any infection and very low for participants with serious infection and exploratory infection episodes. Mortality and heart-failure hospitalisation outcomes were of moderate certainty. All-cause and cardiovascular hospitalisation were rated low certainty because of imprecision and missing results across studies. Domain-specific reasons are given in online supplemental table S4.

### Publication bias

There was no clear evidence of funnel asymmetry for binary all-cause mortality (14 trials; Egger t=−0.12, p=0.903) or participants with heart-failure hospitalisation (10 trials; t=−1.48, p=0.176; online supplemental figures S2 and S3). These tests do not exclude selective reporting, particularly for infection outcomes, for which formal asymmetry testing was not appropriate.

## Discussion

### Principal findings

Three trials reported compatible counts of participants with any infection (OR 0.82, 95% CI 0.64 to 1.08; low certainty), and three reported participants with serious infection (OR 0.83, 0.51 to 1.21; very low certainty). Neither analysis demonstrated a clear increase in infection, but the evidence does not establish comparable risk between treatment groups. Including FAIR-HF2 in a sensitivity analysis shifted the OR to 1.00 and increased I² to 77%; however, its infection definition could not be confirmed. Episode-burden ratios were 1.07 (95% CI 0.76 to 1.86) for infections of any severity and 1.11 (0.82 to 1.42) for serious infections, both with very low certainty. Interpretation was limited by the absence of arm-specific person-time and information on recurrent infections within participants.

Intravenous iron probably reduces total first and recurrent heart-failure hospitalisations. The common-effect estimate indicated a 22% relative reduction over complete follow-up. Analyses of participants with at least one hospitalisation also favoured intravenous iron. All-cause mortality was not clearly altered in either the HR or binary analyses. Quality-of-life findings generally favoured treatment, but differences in instruments, score types and assessment times precluded pooling.

### Relation to existing evidence

Shah and colleagues found an increased infection risk across 154 randomised trials in diverse clinical populations (RR 1.16, 95% CI 1.03 to 1.29).^14^ Differences in formulations, doses, comparators and infection definitions limit direct comparison with the heart-failure evidence. Adverse-event ascertainment was a limitation in both reviews, and the present findings do not establish whether heart failure modifies the effect of intravenous iron on infection risk.

The 2025 heart-failure meta-analysis combined six major trials and used individual-participant data from five, allowing more detailed harmonisation than an aggregate-data analysis.^15^ Its infection OR of 1.02 (95% CI 0.66 to 1.59) was compatible with both benefit and harm. The present analysis extends the assessment of infection reporting across publications, registries and regulatory documents by distinguishing affected participants from episodes, separating infection by severity, and identifying unavailable outcomes. These differences in reporting units and definitions help explain variation between pooled estimates. However, examining additional sources did not resolve the lack of consistent infection definitions or reporting.

Infection reporting varied across trials. IRONMAN reported participants with serious infection and separately adjudicated infection-related clinical endpoints^41^; its median follow-up of 2.7 years was substantially longer than the safety observation periods in FAIR-HF and CONFIRM-HF. FAIR-HF2 reported infection episodes, while participant counts from a secondary source were restricted to sensitivity analysis because their definition could not be confirmed. Higher serious-infection episode ratios in trials with longer follow-up should be interpreted cautiously, as follow-up duration and recruitment setting could not be assessed independently.

The reduction in heart-failure hospitalisations is consistent with the 2025 meta-analysis^15^ and guideline recommendations for intravenous iron in symptomatic heart failure with iron deficiency.^11^ Recurrent-event rate ratios and participant-level risks provide complementary information about repeated admissions and the occurrence of at least one admission, respectively. Agreement between these findings strengthens the evidence for a reduction in hospitalisation. Evidence concerning infection remains of low or very low certainty, and these results do not establish the safety of intravenous iron during active infection or during sepsis.

### Biological and clinical interpretation

Iron may influence infection through competing mechanisms. Sequestration of circulating iron is part of nutritional immunity, and transient non-transferrin-bound iron after infusion could facilitate microbial growth or alter innate immune function.^12 13^ Conversely, iron deficiency may impair T-cell, macrophage and neutrophil function, while newer iron-carbohydrate complexes release labile iron more slowly than older preparations.^13^ The available heart-failure trials do not demonstrate a clear increase in infection, but the sparse and heterogeneous evidence cannot exclude a clinically important effect.

These findings do not establish the safety of intravenous iron during active or uncontrolled infection, after repeated high cumulative exposure, or in substantially immunosuppressed patients. Evidence was concentrated in trials of ferric carboxymaltose and ferric derisomaltose. Treatment decisions should consider infection severity, the urgency of iron replacement and relevant clinical guidance. Evidence concerning anaphylaxis and serious hypersensitivity was insufficient for a comparative estimate.

### Strengths and limitations

Trials were eligible irrespective of whether they reported infection outcomes, and no infection-outcome filter was used in the search. Examination of publications, companion reports, registries and regulatory documents allowed infection outcomes to be classified by severity, reporting unit, analysis population and observation window. Binomial models were used for affected participants, with Poisson models restricted to exploratory analyses of episode counts. Sensitivity analyses examined the influence of individual trials, data sources, follow-up and risk of bias. Mortality was assessed using HRs, while hospitalisation was evaluated separately as recurrent events and as the risk of at least one admission.

The principal limitation is incomplete infection reporting: trials contributing to the primary outcome enrolled only 24% of all randomised participants. Several large trials collected adverse events without reporting a compatible any-infection outcome. Selectively missing results could therefore bias the pooled estimate despite 94% participant coverage in trials contributing to some infection analysis. Infection was usually an adverse event rather than a uniformly adjudicated endpoint; one count was reconstructed from rounded percentages, and FAIR-HF2’s participant-level definition remained unconfirmed. Sparse strata and confounded trial characteristics precluded reliable formulation or setting comparisons. Episode analyses lacked person-time, censoring and recurrence-adjusted variance; registry thresholds and an unresolved FAIR-HFpEF discrepancy prevented pooling of non-serious episodes.

The principal common-effect analyses assumed that intravenous iron had the same relative treatment effect across trials. Random-effects sensitivity analyses allowed treatment effects to differ between trials and produced wider CIs. Binary analyses compared the proportion of participants experiencing an event over different observation periods, without accounting for time to the event or censoring. Restricting these analyses to trials with at least 24 weeks of follow-up excluded shorter studies but did not make follow-up durations comparable or address censoring. Two mortality HRs came from a published meta-analysis rather than original reports, although source restriction gave a similar estimate. Absolute mortality effects depend on the chosen baseline risk and proportional-hazards assumption. CENTRAL was not searched, which may have limited trial identification despite database, registry and regulatory searches. Unpublished data may also remain unavailable; contacted trial authors did not respond. Other recognised adverse effects of intravenous iron were outside the scope of this review.

### Implications for future research

Future heart-failure trials of intravenous iron should prespecify infection as a safety outcome and report both the number of participants with at least one infection and the total number of recurrent events. Arm-specific person-time, time to first infection, infection site and severity, hospitalisation, death, antimicrobial use and microbiological confirmation should be reported using standard definitions. Safety analyses should also record the timing of infection relative to infusion, cumulative iron exposure and achieved ferritin and transferrin saturation. Prespecified analyses by iron formulation, heart-failure phenotype, renal function, diabetes, baseline transferrin saturation and recent or active infection would help identify clinically important effect modification.

## Conclusions

Intravenous iron probably reduces heart-failure related hospitalisations and has little or no effect on all-cause mortality. No clear increase in infection was observed, but low- or very-low-certainty evidence leaves clinically important effects uncertain. Future trials should prespecify standardised infection outcomes, and report affected participants, recurrent events and arm-specific person-time.

## Supplementary material

10.1136/openhrt-2026-004486 online supplemental file 1

## Data Availability

All data relevant to the study are included in the article or uploaded as supplementary information.
