# Supplementary material for: Infection risk and clinical outcomes with intravenous iron in adults with heart failure and iron deficiency: a systematic review and meta-analysis of randomised controlled trials
Source: Open Heart. 2026 Nov 9;13(2):e004486. doi: 10.1136/openhrt-2026-004486 (PMC13756238; doi:10.1136/openhrt-2026-004486)
Supplement: Supplementary file 1 [file openhrt-13-2-s001.docx]

**Infection risk and clinical outcomes with intravenous iron in adults with heart failure and iron deficiency: a systematic review and meta-analysis of randomised controlled trials**

Supplementary material

Supplemental Methods;

Supplemental Tables S1–S11;

Supplemental Figures S1–S11;

Supplemental Appendix 1

# Supplemental Methods

The primary participant-level any-infection synthesis comprised FAIR-HF, AFFIRM-AHF and CONFIRM-HF. Serious-infection participants were analysed separately in FAIR-HF, CONFIRM-HF and IRONMAN. FAIR-HF2 participant counts from the 2025 meta-analysis [15] were used only in a conditional sensitivity analysis because their definition, population and observation window could not be independently matched.

Random-effects models were prioritised for every infection synthesis because infection definitions, severity thresholds, ascertainment and follow-up varied. Binomial-normal GLMMs with a logit link estimated ORs for affected participants. Models had fixed study intercepts, a normally distributed random treatment effect and symmetric treatment coding (+½/−½). Likelihoods were integrated using 60-point Gauss–Hermite quadrature. Heterogeneity was estimated by maximum likelihood, CIs by profile likelihood and P values by likelihood-ratio tests. No continuity correction was used.

Poisson-normal GLMMs with log(participant number) offsets estimated infection episodes per participant over the reporting window. They did not estimate person-time incidence rate ratios. These analyses assume comparable average exposure between trial arms and cannot adjust for censoring, competing death or within-person clustering. Sensitivity analyses for serious episodes inflated each arm’s Poisson log-ratio variance by 1+(episodes/participants)/θ, with θ = 1, 0.5 and 0.25. Smaller θ assumes greater overdispersion; these parameters were not estimated. A 0.5 addition to each event count was used when required for this exploratory inverse-variance calculation.

Common-effect models provided the principal mortality and hospitalisation estimates under an assumed common relative effect within each synthesis. This assumption was based on the clinical interpretation of the endpoint and was not inferred from I². Mantel–Haenszel pooling estimated binary RRs without continuity correction. Mortality HRs and trial-reported recurrent-event rate ratios were pooled by inverse variance on the log scale. Recurrent HF estimates at 12 months and over complete follow-up were kept separate. Four mortality HRs came from original reports; FAIR-HF and AFFIRM-AHF came from the 2025 meta-analysis. A sensitivity analysis was restricted to the four HRs available in original trial reports.

Random-effects sensitivity analyses for clinical outcomes used REML with modified Hartung–Knapp inference (variance factor bounded below at one; k−1 degrees of freedom). The same method was used for infection log-RR sensitivity analyses. Conventional REML–Wald, Mantel–Haenszel and exploratory dispersion models are identified separately in Supplemental Table S5. For inverse-variance log RRs, 0.5 was added to all four cells only when a trial had a zero-event arm. Q and I² used inverse-variance log effects from trials without a zero-event arm; HR and rate-ratio syntheses used reported log effects. Double-zero trials were described without a relative-effect estimate.

Binary mortality and hospitalisation analyses were restricted to outcomes with observation windows of at least 24 weeks in sensitivity analyses. SFGC’s HF readmission counts (4/18 versus 3/16) were reported at six months. Toblli 2015 was excluded from all-cause hospitalisation because its IV-arm numerator was unreported. FERRIC-HF event counts, and Youssef admissions of unspecified cause were included only in a sensitivity analysis. These decisions and trial-level data are provided in Supplemental Tables S7 and S8.

Infection stratification by formulation, recruitment setting, blinding, comparator and reporting window was descriptive. Single-trial strata and confounded characteristics precluded reliable subgroup inference. No interaction tests, meta-regression, infection funnel-asymmetry tests or infection prediction intervals were reported. Low-risk-only analyses excluded results raising some concerns; no infection result was rated high risk. Non-serious registry episodes were not pooled because of reporting thresholds, unresolved overlap and the FAIR-HFpEF discrepancy.

Mortality absolute effects were derived as 1−(1−p)^HR at two illustrative 12-month baseline risks: p = 14/151 (CONFIRM-HF control arm) and 96/550 (AFFIRM-AHF control arm). Applying a pooled HR at 12 months assumes proportional hazards. Other binary absolute effects used the crude pooled control risk and the principal OR or RR. These estimates span trial-specific observation windows and are approximate. Missing results across studies were considered separately from within-trial risk of bias in GRADE.

Egger’s regression was used to assess small-study effects in binary mortality data from 14 trials (t = −0.12, P = 0.903) and HF hospitalisation data from 10 trials (t = −1.48, P = 0.176). Trial-level log RRs with continuity correction for zero-event arms were used in the plots and regression tests.

# Supplemental Table S1: Characteristics and findings of the included trials

| **Study** | **Population and design** | **Intervention and comparator** | **Follow-up** | **Principal relevant findings** | **Infection reporting and use in this review** |
| --- | --- | --- | --- | --- | --- |
| **Toblli et al. (2007) [28]*** | n=40 (20/20). Adults with chronic HFrEF (LVEF ≤35%), CKD and iron-deficiency anaemia; single-centre, double-blind RCT. | Iron sucrose 200 mg weekly for 5 weeks (total 1,000 mg) plus standard care vs saline placebo plus standard care. | 6 months | Iron sucrose improved haemoglobin and iron indices, NYHA class, 6-minute walk distance, quality of life, renal function and inflammatory/cardiac biomarkers. HF hospitalisation occurred in 0/20 vs 5/20 participants. | No extractable infection outcome reported. |
| **FERRIC-HF Okonko et al. (2008) [29]** | n=35 (24/11). Ambulatory symptomatic HFrEF with iron deficiency, with or without anaemia; multicentre, open-label RCT. | Iron sucrose given as repletion and maintenance therapy vs no intravenous iron; background HF therapy continued. | 16 weeks | The prespecified anaemic subgroup had improved peak oxygen uptake per kg; NYHA class and patient global assessment improved overall. The small trial was not designed for hospitalisation or mortality effects. | The primary report lists non-serious pneumonia in 0/24 versus 1/11 patients. This single named outcome was not combined with serious pneumonia or inferred to represent all infections. Coryzal symptoms were excluded as non-specific. |
| **FAIR-HF Anker et al. (2009) [5]** | n=459 (304/155; safety denominators 305/154). Ambulatory NYHA II–III HFrEF with iron deficiency, with or without anaemia; multinational, double-blind RCT. | Ferric carboxymaltose (FCM), up to 200 mg weekly until iron repletion and then every 4 weeks, vs saline placebo. | 24-week treatment; safety outcomes to week 26 | FCM improved patient global assessment, NYHA class, 6-minute walk distance and quality of life. Death and overall serious-adverse-event rates were similar. | Any-infection patients: 50/305 versus 24/154. Serious-infection patients: 2/305 versus 0/154, calculated by summing mutually exclusive anaemia strata in the companion report. Any-infection episodes: 56 versus 32; serious episodes: 2 versus 0. Participant outcomes are shown in Figure 2A/B and episode outcomes in Supplemental Figure S8. |
| **IRON-HF Beck-da-Silva et al. (2013) [30]** | n=23 randomised to intravenous iron, oral iron or placebo; the review comparison used intravenous iron (n=10) vs placebo (n=6). Chronic HFrEF with iron-deficiency anaemia; single-centre RCT. | Iron sucrose vs oral ferrous sulphate vs placebo. The oral-iron arm was not combined with the control arm in this review. | 3 months | Only intravenous iron produced a statistically significant within-group increase in peak oxygen uptake. The trial was stopped early and was markedly underpowered for clinical events. | No extractable infection outcome reported. |
| **CONFIRM-HF Ponikowski et al. (2015) [6]** | n=304 (152/152; analysis population 150/151). Ambulatory symptomatic HFrEF (LVEF ≤45%) with iron deficiency; multinational, double-blind RCT. | FCM dosed according to body weight and haemoglobin, with repeat dosing through 52 weeks as required, vs saline placebo. | 52 weeks | FCM increased 6-minute walk distance by approximately 33 m at week 24, with benefit sustained to week 52; NYHA class, patient global assessment, fatigue and quality of life improved, and first HF hospitalisation was reduced. | CSR synopsis: any-infection patients 40/152 versus 51/152, uniquely reconstructed from 26.3%/33.6%. TGA Table 4 directly reports serious-infection patients 6/152 versus 12/152 and episodes 8 versus 14. Infection TEAEs leading to hospitalisation: 5/152 versus 12/152, reconstructed from 3.3%/7.9%. Registry adverse-event columns are placebo then FCM; all values here are FCM then placebo. Source details: Supplemental Table S6. |
| **Toblli et al. (2015) [31]*** | n=60 (30/30). Adults with chronic HFrEF, CKD and iron-deficiency anaemia; single-centre, double-blind RCT. | Iron sucrose 200 mg weekly for 5 weeks (total 1,000 mg) plus standard care vs saline placebo plus standard care. | 6 months | Iron sucrose improved iron indices, NYHA class, renal function, LVEF and cardiac dimensions and reduced inflammatory markers. The cohort included participants reported in Toblli 2007; the reports did not contribute to the same synthesis. | No extractable infection outcome reported. |
| **EFFECT-HF van Veldhuisen et al. (2017) [32]** | n=172. Ambulatory HFrEF with iron deficiency; multicentre, open-label, blinded-endpoint RCT. | FCM, initially dosed according to weight and haemoglobin and repeated when required, vs standard care without intravenous iron. | 24 weeks | In the prespecified imputed analysis, peak oxygen uptake was maintained with FCM but declined with standard care; the non-imputed sensitivity analysis was not significant. Symptoms and iron indices improved. | EUCTR results give 7 versus 4 serious infection episodes (safety N=88/85) and 19 versus 11 listed non-serious episodes. Serious episodes and exact serious terms were analysed separately. The 26/15 combined total was excluded because overlap between serious and non-serious reporting sections could not be determined. |
| **Youssef et al. (2017) [33]** | n=60 (40/20). Adults admitted with acute decompensated HFrEF (LVEF <40%), NYHA III–IV symptoms and iron-deficiency anaemia; randomised placebo-controlled conference report. | Intravenous iron sucrose vs saline placebo. | 3 months | Haemoglobin improved, but the study did not show a clear reduction in rehospitalisation or mortality. Reporting was limited to a conference abstract. | No extractable infection outcome reported. |
| **PRACTICE-ASIA-HF Yeo et al. (2018) [34]** | n=50 (25/25). Asian patients stabilised after an acute HF admission and found to have iron deficiency; multicentre, double-blind pilot RCT. | Single FCM dose of 1,000 mg vs saline placebo. | 12 weeks | No significant between-group improvement was seen in 6-minute walk distance (adjusted difference 0.88 m, 95% CI −30.2 to 32.0), quality of life or HF admission. | One mild urinary tract infection was mentioned within mixed adverse-event reporting, but the treatment arm was not identifiable; no extractable arm-level infection outcome. |
| **FERRIC-HF II Charles-Edwards et al. (2019) [35]** | n=40 (21/19). Symptomatic chronic HFrEF (LVEF ≤45%) with iron deficiency; double-blind mechanistic RCT. | Single total-dose infusion of iron isomaltoside 1000 (ferric derisomaltose) vs saline placebo. | 2 weeks | Intravenous iron shortened skeletal-muscle phosphocreatine recovery time (adjusted difference −6.8 s), consistent with improved mitochondrial function; dyspnoea improved, whereas haemoglobin did not materially change. | No extractable infection outcome reported. |
| **FCM-HF-IN Dhoot et al. (2020) [36]** | n=70 (35/35). Symptomatic chronic HFrEF with iron deficiency; single-centre, open-label RCT. | FCM in addition to guideline-directed HF treatment vs guideline-directed treatment alone. | 12 weeks | FCM improved peak oxygen uptake, NYHA class, 6-minute walk distance, Minnesota Living with Heart Failure score and iron indices. The trial was small and not powered for hospitalisation or mortality. | No extractable infection outcome reported. |
| **AFFIRM-AHF Ponikowski et al. (2020) [7]** | n=1,132 randomised; modified efficacy population n=1,108. Adults stabilised after acute HF (LVEF <50%) with iron deficiency; multinational, double-blind RCT. | FCM before discharge and at weeks 6, 12 and 24 when iron deficiency persisted vs placebo. | 52 weeks | The primary composite of total HF hospitalisations and cardiovascular death did not reach statistical significance (rate ratio 0.79, 95% CI 0.62–1.01; P=.059); total HF hospitalisations were reduced, with no cardiovascular-mortality effect. | Any-infection patients: 102/559 versus 121/551; any-infection episodes: 143 versus 165. Registry serious-infection episodes: 52 versus 65, summing reported infection terms. EUCTR and ClinicalTrials.gov duplicate the same serious-event records and were included once. Unique serious-infection patients could not be obtained by summing preferred terms. |
| **IRON-CRT Martens et al. (2021) [37]** | n=75 (37/38). HFrEF with iron deficiency and persistent LVEF <45% at least 6 months after cardiac resynchronisation therapy; double-blind RCT. | FCM (mean dose approximately 959 mg) vs placebo. | 3 months | FCM improved LVEF (+4.22% vs −0.23%), left-ventricular end-systolic volume, contractility, peak oxygen uptake and Kansas City Cardiomyopathy Questionnaire score. | At 3 months, Table 2 reports no serious adverse events in either arm (0/37 versus 0/38). Serious infections were therefore classified as absent in both arms during this period. These descriptive data do not establish the absence of infections of any severity. |
| **SFGC pilot Marcusohn et al. (2022) [38]** | n=34 (18/16). Adults hospitalised with acute HF and iron deficiency; prospective, single-blind pilot RCT. | Sodium ferric gluconate complex 125 mg/day for 3–5 doses plus optimal medical therapy vs optimal medical therapy alone. | 3 and 6 months | Recruitment stopped early during the COVID-19 pandemic. No significant between-group difference was shown in 6-minute walk distance, death or HF readmission. | No extractable infection outcome reported. |
| **IRONMAN Kalra et al. (2022) [8]** | n=1,137 (569/568). HFrEF (LVEF ≤45%) with iron deficiency; UK multicentre, open-label, blinded-endpoint RCT. | Ferric derisomaltose, dosed and re-dosed according to weight, haemoglobin and iron indices, vs usual care. | Median 2.7 years | The primary endpoint of recurrent HF hospitalisation and cardiovascular death was not statistically significant in the main analysis (rate ratio 0.82, 95% CI 0.66–1.02; P=.070); a prespecified COVID-19 sensitivity analysis favoured treatment. | Serious-infection patients: 142/559 treated ferric-derisomaltose recipients versus 162/568 usual-care participants; these contribute only to the serious-patient analysis (Figure 2B). Separate adjudicated infection hospitalisation/death outcomes use randomised N=569/568 and trial-reported HRs or recurrent-event rate ratios (Supplemental Figure S8C). |
| **HEART-FID Mentz et al. (2023) [9]** | n=3,065 (1,532/1,533). Ambulatory HFrEF with iron deficiency; multinational, double-blind RCT. | FCM every 6 months when indicated by haemoglobin and iron indices vs placebo. | Median 1.9 years | The hierarchical primary composite did not meet the prespecified significance threshold (win ratio 1.10, 99% CI 0.99–1.23; P=.019 with α=.01). Component differences in death, HF hospitalisation and 6-minute walk distance were modest. | ClinicalTrials.gov results give 267 versus 216 serious infection episodes across 87 reported preferred terms (N=1,532/1,533). Exact serious terms contribute to Supplemental Figure S6. Other-event totals (168/145) were not added because completeness and severity-section overlap were unresolved. Episode totals are not unique patient counts. |
| **FAIR-HFpEF von Haehling et al. (2024) [39]** | n=39 (18/21). Symptomatic HFpEF with iron deficiency; multicentre, double-blind RCT stopped early because of slow recruitment. | FCM vs saline placebo, with repeat dosing according to iron indices. | 24-week efficacy visit; publication safety follow-up to 52 weeks | FCM improved 6-minute walk distance by 49 m (95% CI 5–93; P=.029). Secondary symptom and quality-of-life outcomes were not significantly different; serious adverse events were fewer with FCM. | Publication: 3 versus 0 infectiological episodes in modified ITT N=18/21 over 52-week safety follow-up. Registry: serious pneumonia 1/20 versus 0/22 patients and 1/0 episodes. Source populations were retained separately. Listed non-serious episodes (3/6) were not pooled because a control term reports 4 affected patients but 3 episodes. Sensitivity analyses excluded these records. |
| **FAIR-HF2 Anker et al. (2025) [10]** | n=1,105 (558/547). Stable chronic HFrEF (LVEF ≤45%) with iron deficiency; multinational, investigator-initiated RCT. | FCM, initial total dose up to 2,000 mg followed by 500 mg every 4 months, vs saline placebo. | Median 16.6 months | FCM did not significantly reduce the prespecified primary outcomes after multiplicity control, including first HF hospitalisation/cardiovascular death, total HF hospitalisations or the TSAT <20% subgroup endpoint; quality of life improved. | Primary supplement: any-infection episodes 289 versus 209; serious episodes 103 versus 81. Sex-specific Table S2 corroborates these totals and was not added again. IPD meta-analysis counts 165/558 versus 118/547 [15] specify complete follow-up but were included only in the participant-level sensitivity analysis because the infection definition could not be confirmed. |
| **Sung et al. (2025) [40]** | n=65 (33/32). Acute decompensated HFrEF (LVEF ≤40%) with iron deficiency; single-centre, open-label RCT. | Iron sucrose delivered as two inpatient and two outpatient infusions (mean total dose approximately 709 mg) vs standard care. | 4 weeks; clinical events to 3 months | Iron sucrose improved ferritin, transferrin saturation and Kansas City Cardiomyopathy Questionnaire score (treatment effect +10.6 points). Three-month mortality and HF/all-cause hospitalisation differences were not statistically significant. | No extractable infection outcome reported. |

Toblli 2007 is a subset of Toblli 2015; their data were not combined within a synthesis. Toblli 2007 contributed eligible HF hospitalisation and quality-of-life data. Toblli 2015 was excluded from the all-cause hospitalisation analysis because its IV-iron numerator was unreported. No extractable infection outcome means unreported or unusable, not zero infections. All counts retain the relevant source population.

Notes: Counts are intervention vs comparator unless otherwise stated. “No extractable infection outcome” means that an arm-level infection estimate suitable for this review was unavailable; it does not indicate that no infections occurred. Event counts were not assumed to represent unique participants. AE, adverse event; CI, confidence interval; CKD, chronic kidney disease; COVID-19, coronavirus disease 2019; FCM, ferric carboxymaltose; HF, heart failure; HFpEF, heart failure with preserved ejection fraction; HFrEF, heart failure with reduced ejection fraction; LVEF, left ventricular ejection fraction; NYHA, New York Heart Association; RCT, randomised controlled trial; RR, risk ratio; SAE, serious adverse event; TSAT, transferrin saturation.

**Supplemental Table S2: Study-level risk of bias synopsis**

| **Study** | **D1** | **D2** | **D3** | **D4** | **D5** | **Overall** | **Supporting rationale** |
| --- | --- | --- | --- | --- | --- | --- | --- |
| **Toblli 2007** | Some concerns | Low | Some concerns | Low | Some concerns | Some concerns | Allocation, missing-data and selective-reporting information was incomplete. |
| **FERRIC-HF** | Some concerns | Some concerns | Some concerns | Some concerns | Some concerns | Some concerns | Open-label design and limited methods and reporting prevented low-risk judgements. |
| **FAIR-HF** | Low | Low | Low | Low | Low | Low | Randomised, double-blind trial with sufficiently complete prespecified reporting. |
| **IRON-HF** | Some concerns | Some concerns | Some concerns | Some concerns | Some concerns | Some concerns | Small trial stopped early, with limited information across all domains. |
| **CONFIRM-HF** | Low | Low | Low | Low | Low | Low | Randomised, double-blind trial with sufficiently complete prespecified reporting. |
| **Toblli 2015** | Some concerns | Low | Some concerns | Low | Some concerns | Some concerns | Allocation, missing-data and selective-reporting information was incomplete. |
| **EFFECT-HF** | Low | Some concerns | Some concerns | Low | Some concerns | Some concerns | Open-label treatment and incomplete outcome/reporting information raised concerns. |
| **Youssef 2017** | Some concerns | Some concerns | Some concerns | Some concerns | Some concerns | Some concerns | Conference-only reporting provided insufficient information for low-risk judgements. |
| **PRACTICE-ASIA-HF** | Some concerns | Some concerns | Some concerns | Some concerns | Some concerns | Some concerns | Small pilot trial with limited methodological and safety-reporting detail. |
| **FERRIC-HF II** | Some concerns | Low | Some concerns | Low | Some concerns | Some concerns | Allocation, missing-data and selective-reporting information was incomplete. |
| **FCM-HF-IN** | Some concerns | Some concerns | Some concerns | Some concerns | Some concerns | Some concerns | Small open-label trial with limited protocol and outcome-reporting detail. |
| **AFFIRM-AHF** | Low | Low | Low | Low | Low | Low | Randomised, double-blind trial with sufficiently complete prespecified reporting. |
| **IRON-CRT** | Low | Low | Some concerns | Low | Some concerns | Some concerns | Missing-data and selective-reporting information was incomplete for some outcomes. |
| **SFGC pilot** | Some concerns | Some concerns | Some concerns | Some concerns | Some concerns | Some concerns | Small pilot stopped early during COVID-19, with limited outcome reporting. |
| **IRONMAN** | Low | Some concerns | Low | Low | Low | Some concerns | Open-label treatment raised concern about deviations, although endpoints were blinded. |
| **HEART-FID** | Low | Low | Low | Low | Some concerns | Some concerns | Registry terms supply serious episodes but do not identify unique patients across all infection terms. Outcome-specific infection judgements are provided in Supplemental Table S3. |
| **FAIR-HFpEF** | Low | Low | Some concerns | Low | Some concerns | Some concerns | The small trial stopped early and had incomplete outcome-reporting information. |
| **FAIR-HF2** | Low | Low | Some concerns | Low | Some concerns | Some concerns | Participant counts came from a secondary IPD source. Complete follow-up is specified, but detailed severity and ascertainment matching remain uncertain. |
| **Sung 2025** | Some concerns | Some concerns | Some concerns | Some concerns | Some concerns | Some concerns | Small open-label trial with limited methods and outcome-reporting information. |

D1, randomisation; D2, deviations from intended interventions; D3, missing outcome data; D4, outcome measurement; D5, selection of the reported result. This table summarises study-level judgements. Assessments specific to each infection result and data source are provided in Supplemental Table S3.

# Supplemental Table S3: Outcome-specific risk of bias for infection

| **Trial** | **Outcome** | **D1** | **D2** | **D3** | **D4** | **D5** | **Overall** | **Rationale** |
| --- | --- | --- | --- | --- | --- | --- | --- | --- |
| FAIR-HF | Any/serious infection participants; any/serious episodes | Low | Low | Low | Low | Low | Low | Unblinded injector not involved in care or assessment; safety population. |
| CONFIRM-HF | Participants with any infection | Low | Low | Low | Low | Low | Low | Reconstruction from rounded percentages is an accuracy issue, not selective reporting; handled by sensitivity analysis. |
| CONFIRM-HF | Participants with serious infection; serious episodes; pneumonia | Low | Low | Low | Low | Low | Low | Direct counts; registry lists all SAEs. |
| AFFIRM-AHF | Any-infection participants and episodes; serious episodes; named serious terms | Low | Low | Low | Low | Low | Low | Safety population 1,110 of 1,132 randomised (undosed participants excluded). |
| IRONMAN | Participants with serious infection | Low | Low | Low | Some concerns | Low | Some concerns | Open-label: investigator-reported SAEs could be influenced by knowledge of allocation (D4). |
| EFFECT-HF | Serious episodes; pneumonia | Low | Low | Low | Some concerns | Low | Some concerns | Open-label, standard-care comparator (D4). |
| HEART-FID | Serious episodes; pneumonia | Low | Low | Low | Low | Low | Low | Registry lists all SAEs; collection up to 67.5 months (a follow-up issue, not bias). |
| FAIR-HFpEF | Serious episodes; pneumonia | Low | Low | Some concerns | Low | Some concerns | Some concerns | Registry safety population (20/22) differs from the publication; which population is analysed is unclear. |
| FAIR-HF2 | Participants with any infection (conditional) | Low | Low | Low | Low | Some concerns | Some concerns | Several eligible definitions and windows (serious or any; 12 months or entire follow-up); which was reported is unconfirmed (D5). |
| FAIR-HF2 | Any/serious infection episodes | Low | Low | Low | Low | Low | Low | Primary report. |

Judgements relate to the specified source and outcome. Low-risk-only sensitivity analyses: serious-infection participants excluding IRONMAN, OR 0.60 (0.17–13.33); serious episodes excluding EFFECT-HF and FAIR-HFpEF, ratio 1.09 (0.75–1.39); serious pneumonia excluding the same two trials, OR 1.26 (0.53–2.29). The primary any-infection result was unchanged because all three results were rated low risk. Recorded signalling-question responses are provided in Supplemental Table S11.

CONFIRM-HF counts reconstructed from rounded percentages were examined in sensitivity analysis; reconstruction alone was not considered evidence of selective reporting. FAIR-HF2 participant counts were excluded from the primary analysis because the infection definition could not be confirmed. Differences in safety populations and the absence of person-time were considered separately from within-trial risk of bias.

# Supplemental Table S4: GRADE evidence profile

| **Outcome** | **Studies; participants** | **Principal effect (95% CI)** | **Risk of bias** | **Inconsistency** | **Indirectness** | **Imprecision** | **Missing evidence** | **Certainty** |
| --- | --- | --- | --- | --- | --- | --- | --- | --- |
| All-cause mortality (time to death) | 6 RCTs; 7,175 | HR 0.93 (0.84–1.03) | Not serious: five of six trials double-blind; death is objective in open-label IRONMAN. Two HRs from the 2025 IPD meta-analysis (source issue, not bias); four-original-report pool 0.92 (0.83–1.03). | Not serious: I² = 0%. | Not serious. | Serious (−1): 95% CI 0.84–1.03 includes a 16% reduction and a 3% increase (REML–mHK 0.81–1.06). | Not serious: six major trials contributed HRs; the 14-trial binary analysis was consistent (RR 0.95, 0.88–1.04). The availability record distinguishes unreported outcomes from double-zero trials. | Moderate |
| HF hospitalisation, participants | 10 RCTs; 5,371 | RR 0.89 (0.80–0.99) | Not serious: weight dominated by double-blind trials; EFFECT-HF (open-label) has little weight. | Not serious: I² = 46%; confidence intervals overlap and the heterogeneity arises from small trials. | Not serious. | Serious (−1): common-effect upper limit 0.99; REML–mHK 0.57–1.11 includes no effect. | Not serious: major trials without usable unique-participant counts reported recurrent HF events. This does not prove that missing binary results are unrelated to treatment effects. | Moderate |
| All-cause hospitalisation, participants | 4 RCTs; 1,962 | RR 0.91 (0.84–0.996) | Not serious: open-label IRONMAN carries most weight, but all-cause hospitalisation is an objective outcome. | Not serious: I² = 0%. | Not serious. | Serious (−1): upper limit 0.996; REML–mHK 0.68–1.16. | Serious (−1): reported for 25% of randomised participants; AFFIRM-AHF, HEART-FID and FAIR-HF2 did not report it. | Low |
| CV hospitalisation, participants | 5 RCTs; 2,011 | RR 0.86 (0.76–0.97) | Not serious. | Not serious: I² = 35%; overlapping confidence intervals. | Not serious. | Serious (−1): REML–mHK 0.45–1.17 includes no effect. | Serious (−1): reported for 25% of randomised participants; the large FCM trials (AFFIRM-AHF, HEART-FID, FAIR-HF2) did not report it. | Low |
| Total (first and recurrent) HF hospitalisations | 3 RCTs; 3,350 | Rate ratio 0.78 (0.67–0.90) | Not serious. | Not serious: I² = 0%. | Not serious. | Serious (−1): REML–mHK includes no effect with k = 3. | Not serious. | Moderate |
| Participants with any infection | 3 RCTs; 1,873 | OR 0.82 (0.64–1.08) | Not serious: three double-blind trials judged low risk; the CONFIRM-HF count is reconstructed from rounded percentages (an accuracy issue; excluding it gives OR 0.85, 0.62–1.29). | Not serious: I² = 0%. (Adding FAIR-HF2 raises I² to 77%; its definition is unconfirmed.) | Not serious. | Serious (−1): OR 0.64–1.08; REML–mHK RR 0.57–1.26. | Serious (−1): reported for 24% of randomised participants; most trials collected adverse events but did not report this outcome. | Low |
| Participants with serious infection | 3 RCTs; 1,890 | OR 0.83 (0.51–1.21) | Serious (−1): open-label IRONMAN (some concerns, D4) contributes most participants; excluding it gives OR 0.60 (0.17–13.33). | Not serious: I² = 15% (OR); follow-up differs (26 weeks to 2.7 years). | Not serious. | Serious (−1): OR 0.51–1.21; REML–mHK RR 0.50–1.48. | Serious (−1): reported for 24% of randomised participants; most trials collected adverse events but did not report this outcome. | Very low |
| Serious infection episodes (exploratory) | 7 RCTs; 6,258 | Episode-burden ratio 1.11 (0.82–1.42) | Not serious: low-risk-only pool (5 trials) 1.09 (0.75–1.39). | Serious (−1): ≤52-week windows 0.81 vs >52 weeks 1.24. | Serious (−1): participants used as exposure; person-time and recurrence unavailable. | Serious (−1): 0.82–1.42. | Serious (−1): incomplete and source-dependent reporting; absence of a listed preferred term was not treated as zero. | Very low |
| Any-severity infection episodes (exploratory) | 4 RCTs; 2,713 | Episode-burden ratio 1.07 (0.76–1.86) | Not serious for included source-specific results | Serious (−1): I² = 82%; estimates differ across trials | Serious (−1): participant offset; no person-time or recurrence-adjusted variance | Serious (−1): broad CI includes clinically important benefit and harm | Serious (−1): selectively available episode totals | Very low |

Domain-specific reasons for downgrading are shown; the lowest certainty category is very low. Missing evidence refers to bias from missing results across studies and is not counted again as within-trial risk of bias. Evidence for participants with any infection was rated low certainty: included results were judged at low risk of bias, but imprecision and missing results each warranted one downgrade. FAIR-HF2 was excluded from the primary analysis. Serious-infection participants were downgraded for risk of bias, imprecision and missing evidence. All-cause and cardiovascular hospitalisation were downgraded for imprecision and missing results. Episode analyses are exploratory.

## Absolute effects and alternative inference

| **Outcome** | **REML–mHK sensitivity** | **Baseline risk** | **Absolute effect with principal model** | **Absolute effect with REML–mHK** |
| --- | --- | --- | --- | --- |
| All-cause mortality (time to death) | HR 0.93 (0.81–1.06) | Illustrative 12-month risks (see absolute effect) | 12-month risk 93/1,000 chronic HF (CONFIRM-HF control arm, 52 weeks: 14/151): 6 fewer per 1,000 (14 fewer to 2 more); 12-month risk 175/1,000 after acute HF (AFFIRM-AHF control arm, 52 weeks: 96/550): 12 fewer per 1,000 (26 fewer to 4 more) | 93/1,000: 6 fewer per 1,000 (17 fewer to 5 more); 175/1,000: 12 fewer per 1,000 (30 fewer to 9 more) |
| HF hospitalisation, participants | RR 0.80 (0.57–1.11) | 228 per 1,000 (crude pooled, windows differ) | 25 fewer per 1,000 (45 fewer to 3 fewer) | 46 fewer per 1,000 (97 fewer to 25 more) |
| All-cause hospitalisation, participants | RR 0.89 (0.68–1.16) | 491 per 1,000 (crude pooled, windows differ) | 43 fewer per 1,000 (81 fewer to 2 fewer) | 54 fewer per 1,000 (155 fewer to 77 more) |
| CV hospitalisation, participants | RR 0.73 (0.45–1.17) | 356 per 1,000 (crude pooled, windows differ) | 49 fewer per 1,000 (84 fewer to 10 fewer) | 97 fewer per 1,000 (195 fewer to 59 more) |
| Total (first and recurrent) HF hospitalisations | Rate ratio 0.78 (0.56–1.08) | – | Not calculated: event rates not reported on a common time scale | – |
| Participants with any infection | RR 0.85 (0.57–1.26) (REML–mHK) | 229 per 1,000 | 34 fewer per 1,000 (70 fewer to 13 more) | 34 fewer per 1,000 (97 fewer to 59 more) |
| Participants with serious infection | RR 0.86 (0.50–1.48) (REML–mHK) | 199 per 1,000 | 28 fewer per 1,000 (87 fewer to 32 more) | 27 fewer per 1,000 (99 fewer to 96 more) |

Binary absolute effects are approximate because baseline risks span trial-specific follow-up. Mortality uses two illustrative 12-month risks and assumes proportional hazards. Additional decimal places identify upper confidence limits below one that would otherwise round to 1.00.

# Supplemental Table S5 Infection models and sensitivity analyses

| **Outcome** | **Analysis** | **Model** | **Trials** | **Estimate (95% CI)** | **p** |
| --- | --- | --- | --- | --- | --- |
| Any-infection patients | Primary | Binomial GLMM | 3 | OR 0.82 (0.64–1.08) | 0.107 |
| Any-infection patients | Model sensitivity | REML + modified Hartung–Knapp | 3 | RR 0.85 (0.57–1.26) | 0.216 |
| Any-infection patients | Model sensitivity | REML, Wald | 3 | RR 0.85 (0.71–1.02) | 0.074 |
| Any-infection patients | Model sensitivity | Mantel–Haenszel | 3 | RR 0.85 (0.71–1.02) | 0.083 |
| Any-infection patients | Leave-one-out: omit FAIR-HF 2009 | Binomial GLMM | 2 | OR 0.77 (0.55–1.03) | 0.066 |
| Any-infection patients | Leave-one-out: omit AFFIRM-AHF 2020 | Binomial GLMM | 2 | OR 0.86 (0.52–1.44) | 0.393 |
| Any-infection patients | Leave-one-out: omit CONFIRM-HF 2015 | Binomial GLMM | 2 | OR 0.85 (0.62–1.29) | 0.256 |
| Any-infection patients | Excluded reconstructed CONFIRM-HF count (P008) | Binomial GLMM | 2 | OR 0.85 (0.62–1.29) | 0.256 |
| Any patients, 52 weeks | Nominal 52-week studies (AFFIRM-AHF, CONFIRM-HF) | Binomial GLMM | 2 | OR 0.77 (0.55–1.03) | 0.066 |
| Any patients + FAIR-HF2 | Sensitivity analysis including FAIR-HF2 | Binomial GLMM | 4 | OR 1.00 (0.66–1.48) | 0.989 |
| Any patients + FAIR-HF2 | Leave-one-out: omit FAIR-HF 2009 | Binomial GLMM | 3 | OR 0.98 (0.55–1.68) | 0.928 |
| Any patients + FAIR-HF2 | Leave-one-out: omit AFFIRM-AHF 2020 | Binomial GLMM | 3 | OR 1.11 (0.63–1.80) | 0.621 |
| Any patients + FAIR-HF2 | Leave-one-out: omit CONFIRM-HF 2015 | Binomial GLMM | 3 | OR 1.10 (0.68–1.75) | 0.610 |
| Any patients + FAIR-HF2 | Leave-one-out: omit FAIR-HF2 2025 | Binomial GLMM | 3 | OR 0.82 (0.64–1.08) | 0.107 |
| Serious-infection patients | Primary | Binomial GLMM | 3 | OR 0.83 (0.51–1.21) | 0.162 |
| Serious-infection patients | Model sensitivity | REML + modified Hartung–Knapp | 3 | RR 0.86 (0.50–1.48) | 0.363 |
| Serious-infection patients | Model sensitivity | REML, Wald | 3 | RR 0.86 (0.68–1.10) | 0.243 |
| Serious-infection patients | Model sensitivity | Mantel–Haenszel | 3 | RR 0.87 (0.72–1.05) | 0.153 |
| Serious-infection patients | Leave-one-out: omit FAIR-HF 2009 | Binomial GLMM | 2 | OR 0.82 (0.44–1.14) | 0.136 |
| Serious-infection patients | Leave-one-out: omit CONFIRM-HF 2015 | Binomial GLMM | 2 | OR 0.86 (0.57–2.95) | 0.291 |
| Serious-infection patients | Leave-one-out: omit IRONMAN 2022 | Binomial GLMM | 2 | OR 0.60 (0.17–13.33) | 0.370 |
| Serious patients, no IRONMAN | Excluded long-follow-up IRONMAN (FAIR-HF, CONFIRM-HF) | Binomial GLMM | 2 | OR 0.60 (0.17–13.33) | 0.370 |
| Serious pneumonia | Exploratory | Binomial GLMM | 5 | OR 1.34 (0.69–2.25) | 0.321 |
| Serious pneumonia | Leave-one-out: omit CONFIRM-HF 2015 | Binomial GLMM | 4 | OR 1.36 (0.65–2.66) | 0.321 |
| Serious pneumonia | Leave-one-out: omit EFFECT-HF 2017 | Binomial GLMM | 4 | OR 1.31 (0.61–2.45) | 0.389 |
| Serious pneumonia | Leave-one-out: omit AFFIRM-AHF 2020 | Binomial GLMM | 4 | OR 1.62 (0.93–2.69) | 0.067 |
| Serious pneumonia | Leave-one-out: omit HEART-FID 2023 | Binomial GLMM | 4 | OR 0.89 (0.46–2.29) | 0.715 |
| Serious pneumonia | Leave-one-out: omit FAIR-HFpEF 2024 | Binomial GLMM | 4 | OR 1.30 (0.63–2.16) | 0.397 |
| Serious sepsis | Exploratory | Binomial GLMM | 3 | OR 1.21 (0.37–2.36) | 0.532 |
| Serious sepsis | Leave-one-out: omit EFFECT-HF 2017 | Binomial GLMM | 2 | OR 1.27 (0.47–2.78) | 0.430 |
| Serious sepsis | Leave-one-out: omit AFFIRM-AHF 2020 | Binomial GLMM | 2 | OR 1.51 (0.17–3.82) | 0.282 |
| Serious sepsis | Leave-one-out: omit HEART-FID 2023 | Binomial GLMM | 2 | OR 0.69 (0.07–2.44) | 0.443 |
| Serious urinary tract infection | Exploratory | Binomial GLMM | 3 | OR 1.31 (0.20–2.77) | 0.580 |
| Serious urinary tract infection | Leave-one-out: omit CONFIRM-HF 2015 | Binomial GLMM | 2 | OR 1.50 (0.46–3.59) | 0.295 |
| Serious urinary tract infection | Leave-one-out: omit AFFIRM-AHF 2020 | Binomial GLMM | 2 | OR 1.55 (0.01–15.64) | 0.432 |
| Serious urinary tract infection | Leave-one-out: omit HEART-FID 2023 | Binomial GLMM | 2 | OR 0.56 (0.03–2.60) | 0.351 |
| Serious bacteraemia | Exploratory | Binomial GLMM | 3 | OR 0.66 (0.13–2.94) | 0.521 |
| Serious bacteraemia | Leave-one-out: omit CONFIRM-HF 2015 | Binomial GLMM | 2 | OR 0.80 (0.15–5.76) | 0.733 |
| Serious bacteraemia | Leave-one-out: omit AFFIRM-AHF 2020 | Binomial GLMM | 2 | OR 0.40 (0.02–2.55) | 0.249 |
| Serious bacteraemia | Leave-one-out: omit HEART-FID 2023 | Binomial GLMM | 2 | OR 0.99 (0.01–13.42) | 0.991 |
| Serious septic shock | Exploratory | Binomial GLMM | 2 | OR 1.79 (0.41–7.46) | 0.289 |
| Serious pneumonia | Excluded FAIR-HFpEF | Binomial GLMM | 4 | OR 1.30 (0.63–2.16) | 0.397 |
| Any infection episodes | Exploratory | Poisson GLMM, log(N) offset | 4 | Episode-burden ratio 1.07 (0.76–1.86) | 0.619 |
| Any infection episodes | Model sensitivity | Mantel–Haenszel, N as exposure | 4 | Episode-burden ratio 1.12 (0.98–1.27) | 0.099 |
| Any infection episodes | Leave-one-out: omit FAIR-HF 2009 | Poisson GLMM, log(N) offset | 3 | Episode-burden ratio 1.13 (0.55–6.80) | 0.461 |
| Any infection episodes | Leave-one-out: omit AFFIRM-AHF 2020 | Poisson GLMM, log(N) offset | 3 | Episode-burden ratio 1.29 (0.84–3.18) | 0.153 |
| Any infection episodes | Leave-one-out: omit FAIR-HF2 2025 | Poisson GLMM, log(N) offset | 3 | Episode-burden ratio 0.88 (0.70–1.21) | 0.209 |
| Any infection episodes | Leave-one-out: omit FAIR-HFpEF 2024 | Poisson GLMM, log(N) offset | 3 | Episode-burden ratio 1.04 (0.70–1.47) | 0.772 |
| Serious infection episodes | Exploratory | Poisson GLMM, log(N) offset | 7 | Episode-burden ratio 1.11 (0.82–1.42) | 0.398 |
| Serious infection episodes | Model sensitivity | Mantel–Haenszel, N as exposure | 7 | Episode-burden ratio 1.15 (1.00–1.32) | 0.048 |
| Serious infection episodes | Leave-one-out: omit FAIR-HF 2009 | Poisson GLMM, log(N) offset | 6 | Episode-burden ratio 1.11 (0.79–1.40) | 0.447 |
| Serious infection episodes | Leave-one-out: omit CONFIRM-HF 2015 | Poisson GLMM, log(N) offset | 6 | Episode-burden ratio 1.17 (0.89–1.51) | 0.194 |
| Serious infection episodes | Leave-one-out: omit EFFECT-HF 2017 | Poisson GLMM, log(N) offset | 6 | Episode-burden ratio 1.10 (0.77–1.41) | 0.502 |
| Serious infection episodes | Leave-one-out: omit AFFIRM-AHF 2020 | Poisson GLMM, log(N) offset | 6 | Episode-burden ratio 1.22 (1.00–1.44) | 0.048 |
| Serious infection episodes | Leave-one-out: omit HEART-FID 2023 | Poisson GLMM, log(N) offset | 6 | Episode-burden ratio 1.01 (0.69–1.59) | 0.937 |
| Serious infection episodes | Leave-one-out: omit FAIR-HF2 2025 | Poisson GLMM, log(N) offset | 6 | Episode-burden ratio 1.04 (0.69–1.57) | 0.812 |
| Serious infection episodes | Leave-one-out: omit FAIR-HFpEF 2024 | Poisson GLMM, log(N) offset | 6 | Episode-burden ratio 1.11 (0.80–1.41) | 0.436 |
| Any episodes, 52 weeks | Nominal 52 weeks (AFFIRM-AHF, FAIR-HFpEF) | Poisson GLMM, log(N) offset | 2 | Episode-burden ratio 0.87 (0.63–33.30) | 0.287 |
| Serious infection episodes | Publication/regulatory sources (FAIR-HF, CONFIRM-HF TGA, FAIR-HF2) | Poisson GLMM, log(N) offset | 3 | Episode-burden ratio 1.16 (0.55–2.01) | 0.414 |
| Serious infection episodes | Original publication-only (FAIR-HF, FAIR-HF2) | Poisson GLMM, log(N) offset | 2 | Episode-burden ratio 1.26 (0.79–2.85) | 0.149 |
| Serious infection episodes | Nominal 52 weeks (CONFIRM-HF, AFFIRM-AHF) | Poisson GLMM, log(N) offset | 2 | Episode-burden ratio 0.75 (0.44–1.12) | 0.105 |
| Serious infection episodes | Confirmed windows ≤52 weeks (FAIR-HF, CONFIRM-HF, EFFECT-HF, AFFIRM-AHF) | Poisson GLMM, log(N) offset | 4 | Episode-burden ratio 0.81 (0.57–1.41) | 0.231 |
| Serious infection episodes | Windows >52 weeks (FAIR-HF2, HEART-FID) | Poisson GLMM, log(N) offset | 2 | Episode-burden ratio 1.24 (1.03–1.49) | 0.034 |
| Serious infection episodes | Overdispersion (negative-binomial variance, theta = 1.0) | Inverse variance, assumed overdispersion | 7 | Episode-burden ratio 1.14 (0.99–1.33) | 0.074 |
| Serious infection episodes | Overdispersion (negative-binomial variance, theta = 0.5) | Inverse variance, assumed overdispersion | 7 | Episode-burden ratio 1.14 (0.98–1.33) | 0.098 |
| Serious infection episodes | Overdispersion (negative-binomial variance, theta = 0.25) | Inverse variance, assumed overdispersion | 7 | Episode-burden ratio 1.14 (0.96–1.35) | 0.146 |
| Any-infection participants | Low-risk-only | Binomial random-effects GLMM | 3 | OR 0.82 (0.64–1.08) | 0.107 |
| Serious-infection participants | Low-risk-only; exclude IRONMAN | Binomial random-effects GLMM | 2 | OR 0.60 (0.17–13.33) | 0.370 |
| Serious infection episodes | Low-risk-only; exclude EFFECT-HF and FAIR-HFpEF | Poisson random-effects GLMM, log(N) offset | 5 | Episode-burden ratio 1.09 (0.75–1.39) | — |
| Serious pneumonia | Low-risk-only; exclude EFFECT-HF and FAIR-HFpEF | Binomial random-effects GLMM | 3 | OR 1.26 (0.53–2.29) | — |

GLMM confidence intervals, including those for episode models, were obtained by profile likelihood; p values were obtained by likelihood-ratio tests. RR sensitivity analyses and variance-inflation analyses used the methods specified above. ORs, RRs, episode-burden ratios and HRs were analysed separately. No multiplicity adjustment was applied. Leave-one-out estimates are shown in Supplemental Figure S7.

The GLMM is the principal model for infection; log-RR REML–mHK, conventional REML–Wald, Mantel–Haenszel and assumed-dispersion calculations are sensitivity or model comparisons. ORs, RRs and episode-burden ratios are distinct measures. Dashes indicate unavailable P values for analyses restricted to results at low risk of bias.

# Supplemental Table S6: Infection data and sources

| **Trial** | **Affected patients (IV vs control)** | **Episodes (IV vs control)** | **Population and window** | **Source and locator** | **Extraction decision** |
| --- | --- | --- | --- | --- | --- |
| FAIR-HF | Any: 50/305 vs 24/154 Serious: 2/305 vs 0/154 | Any: 56 vs 32 Serious: 2 vs 0 | As treated, 305/154; 24-week treatment, safety to week 26 | [S1] Table 2, p 2445; [S2] Table 2 and safety Methods | Serious-patient counts combine disjoint anaemia strata. Registry synopsis reports alternative 49 patients/55 events in the IV arm; publication counts were used. |
| CONFIRM-HF | Any: 40/152 vs 51/152 Serious: 6/152 vs 12/152 Hospitalisation-linked: 5/152 vs 12/152 | Serious: 8 vs 14 Listed non-serious: 28 vs 36 (descriptive) | Safety N=152/152; nominal 52-week study, CSR TEAE follow-up rules | [S3] CSR PDF pp 10, 16–17 (printed pp 11, 17–18); [S4] TGA Table 4, p 17; [S5] EUCTR results | Any and hospitalisation-linked patients uniquely reconstructed from 26.3%/33.6% and 3.3%/7.9%. Serious patients directly reported. Registry AE columns are placebo then FCM; counts here are IV then control. |
| EFFECT-HF | Exact serious terms only; no unique all-serious patient union | Serious: 7 vs 4 Listed non-serious: 19 vs 11 (descriptive) | Registry safety N=88/85; week 24 plus spontaneous reports to 30 days after the last visit | [S6] EUCTR adverse events, infection preferred terms | Serious-episode totals were obtained by summing reported terms. Term-specific participant counts were not summed because individuals could have more than one diagnosis. The combined serious/non-serious total of 26/15 was excluded because overlap between sections could not be determined. |
| AFFIRM-AHF | Any: 102/559 vs 121/551 No unique serious patient union recovered | Any: 143 vs 165 Serious: 52 vs 65 | Safety N=559/551; through 52 weeks | [S7] Primary appendix p 27, section 12; [S8] ClinicalTrials.gov serious-AE module | The duplicate EUCTR serious-event dataset was included once. Serious preferred-term patient counts do not define a unique patient union. |
| IRONMAN | Serious: 142/559 vs 162/568; clinical endpoints use N=569/568 | Clinical hospitalisation episodes are separate from all serious infection AEs | Treated safety N=559/568; complete follow-up median 2.7 years | [S9] Primary Table 3, p 2206; [S10] companion Tables 2–3 | Trial-reported HRs and rate ratios were used for clinical endpoints. Main-cause and contributory-cause infection outcomes were kept separate, as were first and recurrent events. |
| HEART-FID | Exact serious terms only; no unique all-infection union | Serious: 267 vs 216; other-event 168/145 not combined | Safety N=1,532/1,533; treatment-period reporting, maximum study duration 67.5 months | [S11] ClinicalTrials.gov adverse-event module | 87 listed serious infection terms. Other-event threshold 1%; section overlap unresolved. Maximum duration is not measured person-time. |
| FAIR-HFpEF | Serious pneumonia: 1/20 vs 0/22 | Publication any: 3 vs 0 Registry serious: 1 vs 0 Listed non-serious: 3 vs 6 (descriptive) | Publication modified ITT N=18/21, safety 52 weeks; registry N=20/22, exact horizon unspecified | [S12] Supplementary Table 3; [S13] EUCTR adverse-event module | Publication and registry populations were analysed separately. A non-serious control term reports 4 affected patients but 3 episodes; non-serious episodes were therefore not pooled. A sensitivity analysis excluded FAIR-HFpEF. |
| FAIR-HF2 | Conditional any: 165/558 vs 118/547 | Any: 289 vs 209 Serious: 103 vs 81 | Randomised N=558/547; complete follow-up, median 16.6 months | [S14] JAMA supplement eTables 7–8; [S15] IPD Supplementary Figure 2; [S16] sex-specific Table S2 | The participant-level construct, population and window in [S15] remain unconfirmed. Counts exceed primary-report serious-episode totals, but this does not establish an any-severity definition. Included only in conditional sensitivity analysis. Sex-stratum episodes reproduce the overall totals and were not added again. AE-table N denotes events, not unique participants. |
| FERRIC-HF | Non-serious pneumonia: 0/24 vs 1/11 | No complete broad total | Source outcome-specific follow-up | [S17] Primary Table 4, p 110 | Descriptive single named infection. Coryzal symptoms excluded; no imputation of all-infection zeros. |
| IRON-CRT | Derived serious-infection zero: 0/37 vs 0/38 | No complete broad total | 3-month adverse-event assessment | [S18] Primary Table 2 | Derived from explicitly zero all-cause serious AEs. Descriptive double-zero only; not evidence of zero infections of any severity. |

Counts are reported as intravenous iron versus control. Participant counts and episodes were analysed separately, and missing data were not imputed as zero. Pooled regulatory safety data from other indications were not attributed to individual heart-failure trials. No trial-specific infection counts were recovered for EFFICACY-HF/FER-CARS-03 or FER-CARS-01; neither trial contributed to the review’s infection analyses.

EMA and FDA searches supplemented data retrieval, but no additional trial-specific infection counts from those agencies were added to the pooled datasets. The CONFIRM-HF regulatory extraction used the TGA report [S4] and CSR [S3]. Searches and documents yielding no usable trial-specific counts were not treated as evidence of zero infections.

## Source references

[S1] Anker SD, Comin Colet J, Filippatos G, Willenheimer R, Dickstein K, Drexler H, Lüscher TF, Bart B, Banasiak W, Niegowska J, Kirwan BA, Mori C, von Eisenhart Rothe B, Pocock SJ, Poole-Wilson PA, Ponikowski P; FAIR-HF Trial Investigators. Ferric carboxymaltose in patients with heart failure and iron deficiency. N Engl J Med. 2009 Dec 17;361(25):2436-48. doi: 10.1056/NEJMoa0908355. Epub 2009 Nov 17. PMID: 19920054.

[S2] Filippatos G, Farmakis D, Colet JC, Dickstein K, Lüscher TF, Willenheimer R, Parissis J, Gaudesius G, Mori C, von Eisenhart Rothe B, Greenlaw N, Ford I, Ponikowski P, Anker SD. Intravenous ferric carboxymaltose in iron-deficient chronic heart failure patients with and without anaemia: a subanalysis of the FAIR-HF trial. Eur J Heart Fail. 2013 Nov;15(11):1267-76. doi: 10.1093/eurjhf/hft099. Epub 2013 Jun 19. PMID: 23787722; PMCID: PMC3806282.

[S3] FER-CARS-05 clinical study report synopsis, 25 November 2014. Public BfArM record. https://portal.dimdi.de/data/ctr/O-2166227-3-0-501E03-20201001082917.pdf

[S4] Therapeutic Goods Administration. Australian Public Assessment Report for ferric carboxymaltose (Ferinject), 1 October 2019. Table 4. https://www.tga.gov.au/sites/default/files/auspar-ferric-carboxymaltose-191001.pdf

[S5] EU Clinical Trials Register. CONFIRM-HF, EudraCT 2011-001695-19. https://www.clinicaltrialsregister.eu/ctr-search/trial/2011-001695-19/results

[S6] EU Clinical Trials Register. EFFECT-HF, EudraCT 2011-000603-40. https://www.clinicaltrialsregister.eu/ctr-search/trial/2011-000603-40/results

[S7] Ponikowski P et al. AFFIRM-AHF. Lancet. 2020;396:1895–1904. Supplementary appendix. https://ars.els-cdn.com/content/image/1-s2.0-S0140673620323394-mmc1.pdf

[S8] ClinicalTrials.gov. AFFIRM-AHF, NCT02937454, posted results. https://clinicaltrials.gov/study/NCT02937454?tab=results

[S9] Kalra PR, Cleland JGF, Petrie MC, Thomson EA, Kalra PA, Squire IB, Ahmed FZ, Al-Mohammad A, Cowburn PJ, Foley PWX, Graham FJ, Japp AG, Lane RE, Lang NN, Ludman AJ, Macdougall IC, Pellicori P, Ray R, Robertson M, Seed A, Ford I; IRONMAN Study Group. Intravenous ferric derisomaltose in patients with heart failure and iron deficiency in the UK (IRONMAN): an investigator-initiated, prospective, randomised, open-label, blinded-endpoint trial. Lancet. 2022 Dec 17;400(10369):2199-2209. doi: 10.1016/S0140-6736(22)02083-9. Epub 2022 Nov 5. PMID: 36347265.

[S10] Foley PW, Kalra PR, Cleland JGF, Petrie MC, Kalra PA, Squire I, Campbell P, Chapman C, Donnelly P, Graham F, Hannah A, Lang NN, Matthews I, Leslie SJ, Pellicori P, Piper S, Ray R, Savage HO, Spencer C, Walsh J, Wong YK, Ford I;  on behalf of the IRONMAN Study Group. Effect of correcting iron deficiency on the risk of serious infection in heart failure: Insights from the IRONMAN trial. Eur J Heart Fail. 2025 Jan;27(1):166-173. doi: 10.1002/ejhf.3504. Epub 2024 Oct 25. PMID: 39453738; PMCID: PMC11798631.

[S11] ClinicalTrials.gov. HEART-FID, NCT03037931, posted results. https://clinicaltrials.gov/study/NCT03037931?tab=results

[S12] von Haehling S, Doehner W, Evertz R, Garfias-Veitl T, Derad C, Diek M, Karakas M, Birkemeyer R, Fillippatos G, Lainscak M, Butler J, Ponikowski P, Böhm M, Friede T, Anker SD. Ferric carboxymaltose and exercise capacity in heart failure with preserved ejection fraction and iron deficiency: the FAIR-HFpEF trial. Eur Heart J. 2024 Oct 5;45(37):3789-3800. doi: 10.1093/eurheartj/ehae479. PMID: 39185895; PMCID: PMC11452748.

[S13] EU Clinical Trials Register. FAIR-HFpEF, EudraCT 2015-005757-12. https://www.clinicaltrialsregister.eu/ctr-search/trial/2015-005757-12/results

[S14] Anker SD, Friede T, Butler J, Talha KM, Placzek M, Diek M, Nosko A, Stas A, Kluge S, Jarczak D, deHeer G, Rybczynski M, Bayés-Genís A, Böhm M, Coats AJS, Edelmann F, Filippatos G, Hasenfuß G, Haverkamp W, Lainscak M, Landmesser U, Macdougall IC, Merkely B, Pieske BM, Pinto FJ, Rassaf T, Visser-Rogers JK, Rosano G, Volterrani M, von Haehling S, Anker MS, Doehner W, Ince H, Koehler F, Savarese G, Khan MS, Rauch-Kröhnert U, Gori T, Trenkwalder T, Akin I, Paitazoglou C, Kobielusz-Gembala I, Kuthi L, Frey N, Licka M, Kääb S, Laugwitz KL, Ponikowski P, Karakas M. Intravenous Ferric Carboxymaltose in Heart Failure With Iron Deficiency: The FAIR-HF2 DZHK05 Randomized Clinical Trial. JAMA. 2025 Jun 10;333(22):1965-1976. doi: 10.1001/jama.2025.3833. PMID: 40159390; PMCID: PMC11955906.

[S15] Anker SD, Karakas M, Mentz RJ, Ponikowski P, Butler J, Khan MS, Talha KM, Kalra PR, Hernandez AF, Mulder H, Rockhold FW, Placzek M, Röver C, Cleland JGF, Friede T. Systematic review and meta-analysis of intravenous iron therapy for patients with heart failure and iron deficiency. Nat Med. 2025 Aug;31(8):2640-2646. doi: 10.1038/s41591-025-03671-1. Epub 2025 Mar 30. PMID: 40159279; PMCID: PMC12353798.

[S16] Karakas M, Friede T, Butler J, Talha KM, Placzek M, Asendorf T, Diek M, Nosko A, Stas A, Kluge S, Jarczak D, DeHeer G, Rybczynski M, Bayes-Genis A, Böhm M, Coats AJS, Edelmann F, Filippatos G, Hasenfuß G, Haverkamp W, Lainscak M, Landmesser U, Macdougall IC, Merkely B, Pieske BM, Pinto FJ, Rassaf T, Visser-Rogers JK, Rosano G, Volterrani M, von Haehling S, Anker MS, Doehner W, Ince H, Koehler F, Savarese G, Khan MS, Kröhnert UR, Gori T, Trenkwalder T, Akin I, Paitazoglou C, Kobielusz-Gembala I, Kuthi L, Frey N, Licka M, Kääb S, Laugwitz KL, Ponikowski P, Anker SD. Intravenous ferric carboxymaltose in heart failure with iron deficiency (FAIR-HF2 DZHK05 trial): Sex-specific outcomes. Eur J Heart Fail. 2025 Nov;27(11):2328-2342. doi: 10.1002/ejhf.3742. Epub 2025 Jul 31. PMID: 40740027; PMCID: PMC12765045.

[S17] Okonko DO, Grzeslo A, Witkowski T, Mandal AK, Slater RM, Roughton M, Foldes G, Thum T, Majda J, Banasiak W, Missouris CG, Poole-Wilson PA, Anker SD, Ponikowski P. Effect of intravenous iron sucrose on exercise tolerance in anemic and nonanemic patients with symptomatic chronic heart failure and iron deficiency FERRIC-HF: a randomized, controlled, observer-blinded trial. J Am Coll Cardiol. 2008 Jan 15;51(2):103-12. doi: 10.1016/j.jacc.2007.09.036. PMID: 18191732.

[S18] Martens P, Dupont M, Dauw J, Nijst P, Herbots L, Dendale P, Vandervoort P, Bruckers L, Tang WHW, Mullens W. The effect of intravenous ferric carboxymaltose on cardiac reverse remodelling following cardiac resynchronization therapy-the IRON-CRT trial. Eur Heart J. 2021 Dec 21;42(48):4905-4914. doi: 10.1093/eurheartj/ehab411. PMID: 34185066; PMCID: PMC8691806.

Registry and public-document retrieval date: 18 October 2026.

# Supplemental Table S7 Clinical outcome models and sensitivity analyses

| **Outcome** | **Dataset** | **Trials** | **IV n/N** | **Control n/N** | **Model** | **Estimate (95% CI)** | **P** | **I² %** |
| --- | --- | --- | --- | --- | --- | --- | --- | --- |
| All-cause hospitalisation | Full follow-up | 4 | 419/1057 | 444/905 | Mantel–Haenszel common effect | 0.9121 (0.8354–0.9957) | 0.0398 | 0.0 |
| All-cause hospitalisation | Full follow-up | 4 | 419/1057 | 444/905 | REML + modified Hartung–Knapp | 0.8899 (0.6846–1.1568) | 0.2520 | 0.0 |
| All-cause hospitalisation | Including FERRIC-HF event counts and Youssef admissions of unspecified cause | 6 | 435/1121 | 457/936 | Mantel–Haenszel common effect | 0.9006 (0.8257–0.9823) | 0.0181 | 0.0 |
| All-cause hospitalisation | Including FERRIC-HF event counts and Youssef admissions of unspecified cause | 6 | 435/1121 | 457/936 | REML + modified Hartung–Knapp | 0.8421 (0.6586–1.0768) | 0.1323 | 0.0 |
| All-cause hospitalisation | ≥24 weeks (FAIR-HF, CONFIRM-HF, IRONMAN) | 3 | 408/1024 | 431/873 | Mantel–Haenszel common effect | 0.9148 (0.8374–0.9994) | 0.0485 | 6.4 |
| All-cause hospitalisation | ≥24 weeks (FAIR-HF, CONFIRM-HF, IRONMAN) | 3 | 408/1024 | 431/873 | REML + modified Hartung–Knapp | 0.8849 (0.5878–1.3323) | 0.3274 | 6.4 |
| HF hospitalisation | Full follow-up (10 trials) | 10 | 536/2766 | 594/2605 | Mantel–Haenszel common effect | 0.8913 (0.8048–0.9870) | 0.0271 | 46.4 |
| HF hospitalisation | Full follow-up (10 trials) | 10 | 536/2766 | 594/2605 | REML + modified Hartung–Knapp | 0.7980 (0.5738–1.1098) | 0.1561 | 46.4 |
| HF hospitalisation | ≥24 weeks (7 trials; SFGC included) | 7 | 524/2671 | 577/2510 | Mantel–Haenszel common effect | 0.8969 (0.8089–0.9945) | 0.0390 | 62.6 |
| HF hospitalisation | ≥24 weeks (7 trials; SFGC included) | 7 | 524/2671 | 577/2510 | REML + modified Hartung–Knapp | 0.7883 (0.4789–1.2977) | 0.2873 | 62.6 |
| Cardiovascular hospitalisation | Full follow-up (5 trials) | 5 | 295/1079 | 332/932 | Mantel–Haenszel common effect | 0.8626 (0.7649–0.9727) | 0.0159 | 35.4 |
| Cardiovascular hospitalisation | Full follow-up (5 trials) | 5 | 295/1079 | 332/932 | REML + modified Hartung–Knapp | 0.7269 (0.4532–1.1659) | 0.1342 | 35.4 |
| Cardiovascular hospitalisation | ≥24 weeks (4 trials) | 4 | 291/1042 | 325/894 | Mantel–Haenszel common effect | 0.8683 (0.7696–0.9798) | 0.0219 | 47.2 |
| Cardiovascular hospitalisation | ≥24 weeks (4 trials) | 4 | 291/1042 | 325/894 | REML + modified Hartung–Knapp | 0.7339 (0.4106–1.3118) | 0.1886 | 47.2 |
| All-cause mortality (binary) | Full follow-up, 14 trials | 14 | 782/3947 | 808/3737 | Mantel–Haenszel common effect | 0.9545 (0.8761–1.0400) | 0.2873 | 0.0 |
| All-cause mortality (binary) | Full follow-up, 14 trials | 14 | 782/3947 | 808/3737 | REML + modified Hartung–Knapp | 0.9565 (0.8705–1.0510) | 0.3265 | 0.0 |
| All-cause mortality (binary) | ≥24 weeks (8 trials) | 8 | 767/3778 | 798/3605 | Mantel–Haenszel common effect | 0.9529 (0.8742–1.0387) | 0.2729 | 0.0 |
| All-cause mortality (binary) | ≥24 weeks (8 trials) | 8 | 767/3778 | 798/3605 | REML + modified Hartung–Knapp | 0.9544 (0.8603–1.0587) | 0.3225 | 0.0 |
| All-cause mortality (HR) | Six trials (four original reports + two meta-analysis-derived HRs) | 6 |  |  | Inverse-variance common effect | 0.9269 (0.8379–1.0253) | 0.1402 | 0.0 |
| All-cause mortality (HR) | Six trials (four original reports + two meta-analysis-derived HRs) | 6 |  |  | REML + modified Hartung–Knapp | 0.9269 (0.8120–1.0580) | 0.2002 | 0.0 |
| All-cause mortality (HR) | Four original-report HRs only | 4 |  |  | Inverse-variance common effect | 0.9202 (0.8255–1.0258) | 0.1335 | 0.0 |
| All-cause mortality (HR) | Four original-report HRs only | 4 |  |  | REML + modified Hartung–Knapp | 0.9202 (0.7715–1.0977) | 0.2305 | 0.0 |

RRs were pooled using Mantel–Haenszel common-effect models without continuity correction; HRs were pooled by inverse variance. Random-effects sensitivity analyses used REML–mHK. The common-effect binary mortality RR was 0.95454 (95% CI 0.87613–1.03996), reported as 0.95 (0.88–1.04); the random-effects estimate rounds to 0.96.

## Mortality HR influence analyses

| **Analysis** | **Model** | **HR (95% CI)** | **P** |
| --- | --- | --- | --- |
| Leave-one-out: omit FAIR-HF | Inverse-variance common effect | 0.929 (0.840–1.028) | 0.154 |
| Leave-one-out: omit FAIR-HF | REML + modified Hartung–Knapp | 0.929 (0.805–1.072) | 0.228 |
| Leave-one-out: omit CONFIRM-HF | Inverse-variance common effect | 0.927 (0.838–1.027) | 0.147 |
| Leave-one-out: omit CONFIRM-HF | REML + modified Hartung–Knapp | 0.927 (0.803–1.071) | 0.221 |
| Leave-one-out: omit AFFIRM-AHF | Inverse-variance common effect | 0.918 (0.824–1.023) | 0.120 |
| Leave-one-out: omit AFFIRM-AHF | REML + modified Hartung–Knapp | 0.918 (0.787–1.070) | 0.195 |
| Leave-one-out: omit IRONMAN | Inverse-variance common effect | 0.919 (0.818–1.033) | 0.157 |
| Leave-one-out: omit IRONMAN | REML + modified Hartung–Knapp | 0.919 (0.780–1.084) | 0.230 |
| Leave-one-out: omit HEART-FID | Inverse-variance common effect | 0.951 (0.829–1.091) | 0.469 |
| Leave-one-out: omit HEART-FID | REML + modified Hartung–Knapp | 0.951 (0.782–1.155) | 0.509 |
| Leave-one-out: omit FAIR-HF2 | Inverse-variance common effect | 0.925 (0.830–1.031) | 0.158 |
| Leave-one-out: omit FAIR-HF2 | REML + modified Hartung–Knapp | 0.925 (0.793–1.079) | 0.231 |

# Supplemental Table S8 Clinical data sources and follow-up

| **Trial** | **Deaths IV** | **Deaths control** | **HR (95% CI)** | **Window** | **Source and locator** | **Source decision** |
| --- | --- | --- | --- | --- | --- | --- |
| FAIR-HF | 5/305 | 4/154 | 0.62 (0.17–2.30) | 24 weeks treatment; safety to 26 weeks | Anker et al. Nature Medicine 2025. Extended Data Figure 5 | HR retained from 2025 meta-analysis Extended Data Figure 5. The HR was not located in the primary report; this estimate was excluded from the original-report restriction. |
| CONFIRM-HF | 12/150 | 14/151 | 0.89 (0.41–1.93) | 52 weeks | Ponikowski et al. 2015, Table 2, p 664 (PDF p 9) | Original report; time to first death; 52 weeks. |
| AFFIRM-AHF | 98/558 | 96/550 | 0.99 (0.75–1.31) | 52 weeks | Anker et al. Nature Medicine 2025. Extended Data Figure 5 | HR retained from 2025 meta-analysis Extended Data Figure 5. The HR was not located in the primary report; this estimate was excluded from the original-report restriction. |
| IRONMAN | 184/569 | 193/568 | 0.95 (0.78–1.17) | Median 2.7 years | Kalra et al. Lancet 2022, Table 2, p 2204 (PDF p 6) | Original report, complete follow-up. The 2025 meta-analysis gives upper CI 1.16; original report gives 1.17. Main-trial deaths are 184 vs 193. |
| HEART-FID | 361/1532 | 376/1533 | 0.90 (0.78–1.05) | Median 1.9 years | Mentz et al. NEJM 2023, Safety, p 980 (PDF p 6) | Original report, complete follow-up, prespecified exploratory all-cause death outcome. Narrative gives 95% CI 0.78-1.05; The 96% CIs for other Table 2 outcomes were not used. |
| FAIR-HF2 | 104/558 | 111/547 | 0.94 (0.72–1.24) | Within 36 months; original trial safety outcome | Anker et al. JAMA 2025, Table 2 and Safety, pp 1969-1971 (PDF pp 5-7) | Original all-cause mortality within 36 months. The 2025 meta-analysis displays 0.90 (0.72-1.14); source estimates are kept separate. |

Original-report HRs: CONFIRM-HF [6], IRONMAN [8], HEART-FID [9] and FAIR-HF2 [10]. FAIR-HF and AFFIRM-AHF HRs were retained from Extended Data Figure 5 of the 2025 meta-analysis [15]. For FAIR-HF2, the original 0.94 (0.72–1.24) was used; the secondary-source 0.90 (0.72–1.14) was not substituted. HR and RR estimates from the same trial were never combined in one synthesis.

## Binary mortality availability

| **Trial** | **IV deaths/N** | **Control deaths/N** | **Window** | **Decision** |
| --- | --- | --- | --- | --- |
| FERRIC-HF | 1/24 | 0/11 | <24 weeks | Included in full-follow-up binary pool |
| FAIR-HF | 5/305 | 4/154 | ≥24 weeks | Included in full-follow-up binary pool |
| IRON-HF | 2/10 | 1/6 | <24 weeks | Included in full-follow-up binary pool |
| CONFIRM-HF | 12/150 | 14/151 | ≥24 weeks | Included in full-follow-up binary pool |
| EFFECT-HF | 0/88 | 4/86 | ≥24 weeks | Included in full-follow-up binary pool |
| Youssef | 6/40 | 3/20 | <24 weeks | Included in full-follow-up binary pool |
| PRACTICE-ASIA-HF | 1/25 | 0/25 | <24 weeks | Included in full-follow-up binary pool |
| AFFIRM-AHF | 98/558 | 96/550 | ≥24 weeks | Included in full-follow-up binary pool |
| IRON-CRT | 0/37 | 3/38 | <24 weeks | Included in full-follow-up binary pool |
| SFGC | 3/18 | 0/16 | ≥24 weeks | Included in full-follow-up binary pool |
| IRONMAN | 184/569 | 193/568 | ≥24 weeks | Included in full-follow-up binary pool |
| HEART-FID | 361/1532 | 376/1533 | ≥24 weeks | Included in full-follow-up binary pool |
| FAIR-HF2 | 104/558 | 111/547 | ≥24 weeks | Included in full-follow-up binary pool |
| Sung | 5/33 | 3/32 | <24 weeks | Included in full-follow-up binary pool |
| FAIR-HFpEF | 0/18 | 0/21 | 52 weeks | Explicitly no deaths with complete survival ascertainment; double-zero, described without a relative-effect estimate |
| FCM-HF-IN | Not tabulated by arm | Not tabulated by arm | 12 weeks | Narrative states no mortality during the study, without an arm-specific mortality table; described only, with no imputed arm counts. |
| Toblli 2007/2015 | Not reported | Not reported | 6 months | No extractable arm-specific death counts identified in the reviewed primary reports; not imputed as zero; overlapping reports |
| FERRIC-HF II | Not reported | Not reported | 2 weeks | No extractable arm-specific mortality comparison identified; not imputed as zero |

The 14-trial relative-effect dataset excludes double-zero comparisons. FAIR-HFpEF explicitly reported complete survival ascertainment through week 52 with no deaths (original Safety section). FCM-HF-IN stated narratively that there was no mortality during the study; arm-specific death counts were not tabulated and were not imputed. Unreported mortality was distinguished from explicit zero-event statements.

## Hospitalisation data and inclusion decisions

| **Outcome** | **Trial** | **IV n/N** | **Control n/N** | **Reporting window** | **Inclusion decision** | **Source and locator** |
| --- | --- | --- | --- | --- | --- | --- |
| HF hospitalisation | Toblli 2007 | 0/20 | 5/20 | 6 months | Included | Toblli et al. 2007, p 1659 and Table 2 (PDF pp 3-4); https://doi.org/10.1016/j.jacc.2007.07.029 |
| HF hospitalisation | FAIR-HF | 6/305 | 7/154 | 24 weeks treatment; safety to 26 weeks | Included | Anker et al. 2009, Table 2, p 2445 (PDF p 10); https://doi.org/10.1056/NEJMoa0908355 |
| HF hospitalisation | CONFIRM-HF | 10/150 | 25/151 | 52 weeks | Included | Ponikowski et al. 2015, Table 2, p 664 (PDF p 9); https://doi.org/10.1093/eurheartj/ehu385 |
| HF hospitalisation | EFFECT-HF | 11/88 | 6/86 | 24 weeks | Included | van Veldhuisen et al. 2017, Results/Table 2, p 1379 (PDF p 6); https://doi.org/10.1161/CIRCULATIONAHA.117.027497 |
| HF hospitalisation | PRACTICE-ASIA-HF | 5/25 | 5/25 | 12 weeks | Excluded <24 weeks | Chua et al. 2018, Table 6 and adverse-event text, p 349 (PDF p 6); https://doi.org/10.1002/ehf2.12250 |
| HF hospitalisation | AFFIRM-AHF | 142/558 | 178/550 | 52 weeks | Included | Ponikowski et al. 2020, Figure 3, p 1900 (PDF p 6); https://doi.org/10.1016/S0140-6736(20)32339-4 |
| HF hospitalisation | IRON-CRT | 1/37 | 4/38 | 3 months | Excluded <24 weeks | Martens et al. 2021, Table 2, p 4912 (PDF p 9); https://doi.org/10.1093/eurheartj/ehab411 |
| HF hospitalisation | SFGC pilot 2022 | 4/18 | 3/16 | 6 months (24-week scheduled assessment) | Included | Marcusohn et al. 2022, Table 1, p 195 (PDF p 2); https://doi.org/10.1097/FJC.0000000000001287 |
| HF hospitalisation | HEART-FID | 351/1532 | 353/1533 | Complete trial follow-up; median 1.9 years | Included | Mentz et al. 2023, Table 2, p 981 (PDF p 7); https://doi.org/10.1056/NEJMoa2304968 |
| HF hospitalisation | Sung 2025 | 6/33 | 8/32 | 3 months clinical events | Excluded <24 weeks | Sung et al. 2025, three-month clinical outcomes; https://doi.org/10.31083/RCM28216 |
| All-cause hospitalisation | FERRIC-HF | 3/24 | 3/11 | 18-week observation; 16-week treatment | Excluded: <24 weeks / events | Okonko et al. 2008, p 108 and Table 4 (PDF p 6); https://doi.org/10.1016/j.jacc.2007.09.036 |
| All-cause hospitalisation | FAIR-HF | 25/305 | 17/154 | 24 weeks treatment; safety to 26 weeks | Included | Anker et al. 2009, Table 2, p 2445 (PDF p 10); https://doi.org/10.1056/NEJMoa0908355 |
| All-cause hospitalisation | CONFIRM-HF | 32/150 | 44/151 | 52 weeks | Included | Ponikowski et al. 2015, Table 2, p 664 (PDF p 9); https://doi.org/10.1093/eurheartj/ehu385 |
| All-cause hospitalisation | Toblli 2015 | Not reported | 8/30 | 6 months | Excluded: numerator unreported | Toblli et al. 2015, Results, p 690 (PDF p 4); https://doi.org/10.1016/j.hlc.2014.12.161 |
| All-cause hospitalisation | Youssef 2017 | 13/40 | 10/20 | 3 months | Excluded: <24 weeks / cause | Youssef et al. 2017, abstract P5825; https://doi.org/10.1093/eurheartj/ehx493.P5825 |
| All-cause hospitalisation | IRONMAN | 351/569 | 370/568 | Median 2.7 years | Included | Kalra et al. 2022, Table 2, p 2204 (PDF p 6); https://doi.org/10.1016/S0140-6736(22)02083-9 |
| All-cause hospitalisation | Sung 2025 | 11/33 | 13/32 | 3 months clinical events | Excluded <24 weeks | Sung et al. 2025, three-month clinical outcomes; https://doi.org/10.31083/RCM28216 |
| Cardiovascular hospitalisation | FAIR-HF | 15/305 | 14/154 | 24 weeks treatment; safety to 26 weeks | Included | Anker et al. 2009, Table 2, p 2445 (PDF p 10); https://doi.org/10.1056/NEJMoa0908355 |
| Cardiovascular hospitalisation | CONFIRM-HF | 21/150 | 33/151 | 52 weeks | Included | Ponikowski et al. 2015, Table 2, p 664 (PDF p 9); https://doi.org/10.1093/eurheartj/ehu385 |
| Cardiovascular hospitalisation | IRON-CRT | 4/37 | 7/38 | 3 months | Excluded <24 weeks | Martens et al. 2021, Table 2, p 4912 (PDF p 9); https://doi.org/10.1093/eurheartj/ehab411 |
| Cardiovascular hospitalisation | IRONMAN | 254/569 | 273/568 | Median 2.7 years | Included | Kalra et al. 2022, Table 2, p 2204 (PDF p 6); https://doi.org/10.1016/S0140-6736(22)02083-9 |
| Cardiovascular hospitalisation | FAIR-HFpEF | 1/18 | 5/21 | 52-week safety surveillance; 24-week efficacy trial | Included | von Haehling et al. 2024, Safety, p 3794 (PDF p 10); https://doi.org/10.1093/eurheartj/ehae479 |

# Supplemental Table S9 Infection subgroup feasibility and descriptive estimates

| **Infection synthesis** | **Characteristic** | **Available levels and trials** | **Feasibility** |
| --- | --- | --- | --- |
| Participants with any infection (primary, 3 trials) | Formulation | All: Ferric carboxymaltose | No: no variation |
| Participants with any infection (primary, 3 trials) | Setting | Chronic (2: FAIR-HF, CONFIRM-HF); Acute/recently decompensated (1: AFFIRM-AHF) | Descriptive only: single-trial stratum |
| Participants with any infection (primary, 3 trials) | Blinding | All: Double-blind | No: no variation |
| Participants with any infection (primary, 3 trials) | Comparator | All: Placebo | No: no variation |
| Participants with any infection (primary, 3 trials) | Follow-up | All: ≤52 weeks | No: no variation |
| Participants with serious infection (3 trials) | Formulation | Ferric carboxymaltose (2: FAIR-HF, CONFIRM-HF); Ferric derisomaltose (1: IRONMAN) | Descriptive only: single-trial stratum; fully confounded with setting, blinding, comparator, follow-up |
| Participants with serious infection (3 trials) | Setting | Chronic (2: FAIR-HF, CONFIRM-HF); Mixed (1: IRONMAN) | Descriptive only: single-trial stratum; fully confounded with formulation, blinding, comparator, follow-up |
| Participants with serious infection (3 trials) | Blinding | Double-blind (2: FAIR-HF, CONFIRM-HF); Open-label (1: IRONMAN) | Descriptive only: single-trial stratum; fully confounded with formulation, setting, comparator, follow-up |
| Participants with serious infection (3 trials) | Comparator | Placebo (2: FAIR-HF, CONFIRM-HF); Usual/standard care (1: IRONMAN) | Descriptive only: single-trial stratum; fully confounded with formulation, setting, blinding, follow-up |
| Participants with serious infection (3 trials) | Follow-up | ≤52 weeks (2: FAIR-HF, CONFIRM-HF); >52 weeks (1: IRONMAN) | Descriptive only: single-trial stratum; fully confounded with formulation, setting, blinding, comparator |
| Any-severity infection episodes (4 trials) | Formulation | All: Ferric carboxymaltose | No: no variation |
| Any-severity infection episodes (4 trials) | Setting | Chronic (3: FAIR-HF, FAIR-HF2, FAIR-HFpEF); Acute/recently decompensated (1: AFFIRM-AHF) | Descriptive only: single-trial stratum |
| Any-severity infection episodes (4 trials) | Blinding | All: Double-blind | No: no variation |
| Any-severity infection episodes (4 trials) | Comparator | All: Placebo | No: no variation |
| Any-severity infection episodes (4 trials) | Follow-up | ≤52 weeks (3: FAIR-HF, AFFIRM-AHF, FAIR-HFpEF); >52 weeks (1: FAIR-HF2) | Descriptive only: single-trial stratum |
| Serious infection episodes (7 trials) | Formulation | All: Ferric carboxymaltose | No: no variation |
| Serious infection episodes (7 trials) | Setting | Chronic (6: FAIR-HF, CONFIRM-HF, EFFECT-HF, HEART-FID, FAIR-HF2, FAIR-HFpEF); Acute/recently decompensated (1: AFFIRM-AHF) | Descriptive only: single-trial stratum |
| Serious infection episodes (7 trials) | Blinding | Double-blind (6: FAIR-HF, CONFIRM-HF, AFFIRM-AHF, HEART-FID, FAIR-HF2, FAIR-HFpEF); Open-label (1: EFFECT-HF) | Descriptive only: single-trial stratum; fully confounded with comparator |
| Serious infection episodes (7 trials) | Comparator | Placebo (6: FAIR-HF, CONFIRM-HF, AFFIRM-AHF, HEART-FID, FAIR-HF2, FAIR-HFpEF); Usual/standard care (1: EFFECT-HF) | Descriptive only: single-trial stratum; fully confounded with blinding |
| Serious infection episodes (7 trials) | Follow-up | ≤52 weeks (4: FAIR-HF, CONFIRM-HF, EFFECT-HF, AFFIRM-AHF); >52 weeks (2: HEART-FID, FAIR-HF2) | Descriptive only: ≤3 trials per stratum |
| Serious pneumonia (5 trials) | Formulation | All: Ferric carboxymaltose | No: no variation |
| Serious pneumonia (5 trials) | Setting | Chronic (4: CONFIRM-HF, EFFECT-HF, HEART-FID, FAIR-HFpEF); Acute/recently decompensated (1: AFFIRM-AHF) | Descriptive only: single-trial stratum |
| Serious pneumonia (5 trials) | Blinding | Double-blind (4: CONFIRM-HF, AFFIRM-AHF, HEART-FID, FAIR-HFpEF); Open-label (1: EFFECT-HF) | Descriptive only: single-trial stratum; fully confounded with comparator |
| Serious pneumonia (5 trials) | Comparator | Placebo (4: CONFIRM-HF, AFFIRM-AHF, HEART-FID, FAIR-HFpEF); Usual/standard care (1: EFFECT-HF) | Descriptive only: single-trial stratum; fully confounded with blinding |
| Serious pneumonia (5 trials) | Follow-up | ≤52 weeks (3: CONFIRM-HF, EFFECT-HF, AFFIRM-AHF); >52 weeks (1: HEART-FID) | Descriptive only: single-trial stratum |

IRONMAN recruitment was mixed outpatient and hospital-based. FAIR-HFpEF registry outcomes were excluded from reporting-window strata because the precise registry horizon was unconfirmed. No subgroup-difference tests were performed; none of these comparisons supports a causal inference about effect modification.

| **Infection synthesis** | **Characteristic** | **Stratum** | **Trials** | **Measure** | **Estimate (95% CI)** | **Model** |
| --- | --- | --- | --- | --- | --- | --- |
| Participants with any infection (primary, 3 trials) | Setting | Chronic | 2 | Odds ratio | 0.86 (0.52–1.44) | GLMM |
| Participants with any infection (primary, 3 trials) | Setting | Acute/recently decompensated | 1 | Odds ratio | 0.79 (0.59–1.06) | single trial |
| Participants with serious infection (3 trials) | Formulation | Ferric carboxymaltose | 2 | Odds ratio | 0.60 (0.17–13.33) | GLMM |
| Participants with serious infection (3 trials) | Formulation | Ferric derisomaltose | 1 | Odds ratio | 0.85 (0.66–1.11) | single trial |
| Participants with serious infection (3 trials) | Setting | Chronic | 2 | Odds ratio | 0.60 (0.17–13.33) | GLMM |
| Participants with serious infection (3 trials) | Setting | Mixed | 1 | Odds ratio | 0.85 (0.66–1.11) | single trial |
| Participants with serious infection (3 trials) | Blinding | Double-blind | 2 | Odds ratio | 0.60 (0.17–13.33) | GLMM |
| Participants with serious infection (3 trials) | Blinding | Open-label | 1 | Odds ratio | 0.85 (0.66–1.11) | single trial |
| Participants with serious infection (3 trials) | Comparator | Placebo | 2 | Odds ratio | 0.60 (0.17–13.33) | GLMM |
| Participants with serious infection (3 trials) | Comparator | Usual/standard care | 1 | Odds ratio | 0.85 (0.66–1.11) | single trial |
| Participants with serious infection (3 trials) | Follow-up | ≤52 weeks | 2 | Odds ratio | 0.60 (0.17–13.33) | GLMM |
| Participants with serious infection (3 trials) | Follow-up | >52 weeks | 1 | Odds ratio | 0.85 (0.66–1.11) | single trial |
| Any-severity infection episodes (4 trials) | Setting | Chronic | 3 | Episode-burden ratio | 1.29 (0.84–3.18) | GLMM |
| Any-severity infection episodes (4 trials) | Setting | Acute/recently decompensated | 1 | Episode-burden ratio | 0.85 (0.68–1.07) | single trial |
| Any-severity infection episodes (4 trials) | Follow-up | ≤52 weeks | 3 | Episode-burden ratio | 0.88 (0.70–1.21) | GLMM |
| Any-severity infection episodes (4 trials) | Follow-up | >52 weeks | 1 | Episode-burden ratio | 1.36 (1.14–1.62) | single trial |
| Serious infection episodes (7 trials) | Setting | Chronic | 6 | Episode-burden ratio | 1.22 (1.00–1.44) | GLMM |
| Serious infection episodes (7 trials) | Setting | Acute/recently decompensated | 1 | Episode-burden ratio | 0.79 (0.55–1.14) | single trial |
| Serious infection episodes (7 trials) | Blinding | Double-blind | 6 | Episode-burden ratio | 1.10 (0.77–1.41) | GLMM |
| Serious infection episodes (7 trials) | Blinding | Open-label | 1 | Episode-burden ratio | 1.69 (0.49–5.77) | single trial |
| Serious infection episodes (7 trials) | Comparator | Placebo | 6 | Episode-burden ratio | 1.10 (0.77–1.41) | GLMM |
| Serious infection episodes (7 trials) | Comparator | Usual/standard care | 1 | Episode-burden ratio | 1.69 (0.49–5.77) | single trial |
| Serious infection episodes (7 trials) | Follow-up | ≤52 weeks | 4 | Episode-burden ratio | 0.81 (0.57–1.41) | GLMM |
| Serious infection episodes (7 trials) | Follow-up | >52 weeks | 2 | Episode-burden ratio | 1.24 (1.03–1.49) | GLMM |
| Serious pneumonia (5 trials) | Setting | Chronic | 4 | Odds ratio | 1.62 (0.93–2.69) | GLMM |
| Serious pneumonia (5 trials) | Setting | Acute/recently decompensated | 1 | Odds ratio | 0.72 (0.33–1.58) | single trial |
| Serious pneumonia (5 trials) | Blinding | Double-blind | 4 | Odds ratio | 1.31 (0.61–2.45) | GLMM |
| Serious pneumonia (5 trials) | Blinding | Open-label | 1 | Odds ratio | 1.47 (0.24–8.99) | single trial |
| Serious pneumonia (5 trials) | Comparator | Placebo | 4 | Odds ratio | 1.31 (0.61–2.45) | GLMM |
| Serious pneumonia (5 trials) | Comparator | Usual/standard care | 1 | Odds ratio | 1.47 (0.24–8.99) | single trial |
| Serious pneumonia (5 trials) | Follow-up | ≤52 weeks | 3 | Odds ratio | 0.83 (0.42–1.96) | GLMM |
| Serious pneumonia (5 trials) | Follow-up | >52 weeks | 1 | Odds ratio | 1.65 (1.08–2.54) | single trial |

# Supplemental Table S10 Representativeness of infection-reporting cohorts

| **Characteristic** | **Primary any-infection analysis** | **Any infection analysis** | **No pooled infection analysis** | **All cohorts** |
| --- | --- | --- | --- | --- |
| Cohorts | 3 | 8 | 10 | 18 |
| Participants in source-reported cohorts | 1,895 | 7,413 | 512 | 7,925 |
| Median sample size (IQR) | 459 (382–796) | 782 (271–1,133) | 55 (36–64) | 68 (42–420) |
| Median publication year (range) | 2015 (2009–2020) | 2021 (2009–2025) | 2018 (2007–2025) | 2019 (2007–2025) |
| Ferric carboxymaltose | 3/3 | 7/8 | 3/10 | 10/18 |
| Ferric derisomaltose | 0/3 | 1/8 | 1/10 | 2/18 |
| Iron sucrose or SFGC | 0/3 | 0/8 | 6/10 | 6/18 |
| Acute or recently decompensated HF | 1/3 | 1/8 | 4/10 | 5/18 |
| Mixed outpatient/hospital recruitment (IRONMAN) | 0/3 | 1/8 | 0/10 | 1/18 |
| Placebo comparator | 3/3 | 6/8 | 6/10 | 12/18 |
| Double-blind | 3/3 | 6/8 | 5/10 | 11/18 |
| Median follow-up, weeks (IQR) | 52 (38–52) | 52 (24–79) | 13 (12–15) | 24 (13–46) |
| Follow-up ≥52 weeks | 2/3 | 5/8 | 0/10 | 5/18 |
| Share of all randomised participants | 24% | 94% | 6% | 100% |

The primary any-infection analysis included FAIR-HF, CONFIRM-HF and AFFIRM-AHF. At least one pooled infection analysis also included EFFECT-HF, IRONMAN, HEART-FID, FAIR-HF2 and FAIR-HFpEF. Cohorts were counted once, including the overlapping Toblli reports. Coverage was calculated from source-reported cohort sizes, which can differ from total enrolment (EFFECT-HF: 172 in the principal analysis, 174 enrolled). Infection models used source-specific safety populations. Trials contributing to at least one infection analysis represented 94% of participants; those contributing to the primary any-infection analysis represented 24%.

# Supplemental Table S11 Recorded RoB 2 signalling responses

Y, yes; PY, probably yes; PN, probably no; N, no; NI, no information; NA, not applicable; NR, not recorded. Domain judgements and supporting rationales are provided in Supplemental Table S3. Unrecorded conditional responses limit reproducibility of these assessments. In FAIR-HFpEF, the recorded response to question 1.3 (PY) is inconsistent with the low-risk D1 judgement; no supporting explanation for this departure was documented.

## Randomisation and deviations

| **Trial** | **Outcome** | **1.1** | **1.2** | **1.3** | **2.1** | **2.2** | **2.3** | **2.6** |
| --- | --- | --- | --- | --- | --- | --- | --- | --- |
| FAIR-HF | Participants with any / serious infection | Y | Y | N | N | PN | NA | Y |
| CONFIRM-HF | Participants with any infection | Y | Y | N | N | PN | NA | Y |
| CONFIRM-HF | Participants with serious infection; serious episodes; pneumonia | Y | Y | N | N | PN | NA | Y |
| AFFIRM-AHF | Participants with any infection; serious episodes; pneumonia | Y | Y | N | N | PN | NA | Y |
| IRONMAN | Participants with serious infection | Y | Y | N | Y | Y | PN | Y |
| EFFECT-HF | Serious episodes; pneumonia | Y | Y | N | Y | Y | PN | Y |
| HEART-FID | Serious episodes; pneumonia | Y | Y | N | N | PN | NA | Y |
| FAIR-HFpEF | Serious episodes; pneumonia | Y | Y | PN | N | PN | NA | PY |
| FAIR-HF2 | Participants with any infection (conditional) | Y | Y | N | N | PN | NA | Y |
| FAIR-HF2 | Serious episodes | Y | Y | N | N | PN | NA | Y |

## Missing data and outcome measurement

| **Trial** | **Outcome** | **3.1** | **4.1** | **4.2** | **4.3** | **4.4** | **4.5** |
| --- | --- | --- | --- | --- | --- | --- | --- |
| FAIR-HF | Participants with any / serious infection | Y | N | N | N | NA | NA |
| CONFIRM-HF | Participants with any infection | Y | N | N | N | NA | NA |
| CONFIRM-HF | Participants with serious infection; serious episodes; pneumonia | Y | N | N | N | NA | NA |
| AFFIRM-AHF | Participants with any infection; serious episodes; pneumonia | PY | N | N | N | NA | NA |
| IRONMAN | Participants with serious infection | Y | N | PN | Y | PY | PN |
| EFFECT-HF | Serious episodes; pneumonia | Y | N | PN | Y | PY | PN |
| HEART-FID | Serious episodes; pneumonia | Y | N | N | N | NA | NA |
| FAIR-HFpEF | Serious episodes; pneumonia | NI | N | N | N | NA | NA |
| FAIR-HF2 | Participants with any infection (conditional) | Y | N | N | N | NA | NA |
| FAIR-HF2 | Serious episodes | Y | N | N | N | NA | NA |

## Selection of the reported result

| **Trial** | **Outcome** | **5.1** | **5.2** | **5.3** |
| --- | --- | --- | --- | --- |
| FAIR-HF | Participants with any / serious infection | Y | N | N |
| CONFIRM-HF | Participants with any infection | Y | N | N |
| CONFIRM-HF | Participants with serious infection; serious episodes; pneumonia | Y | N | N |
| AFFIRM-AHF | Participants with any infection; serious episodes; pneumonia | Y | N | N |
| IRONMAN | Participants with serious infection | Y | N | N |
| EFFECT-HF | Serious episodes; pneumonia | Y | N | N |
| HEART-FID | Serious episodes; pneumonia | Y | N | N |
| FAIR-HFpEF | Serious episodes; pneumonia | Y | N | PY |
| FAIR-HF2 | Participants with any infection (conditional) | PY | PY | N |
| FAIR-HF2 | Serious episodes | Y | N | N |

Question key: 1.1 random allocation sequence; 1.2 concealment; 1.3 baseline imbalances suggesting a randomisation problem; 2.1 participant awareness; 2.2 personnel awareness; 2.3 deviations arising from trial context; 2.6 appropriate analysis; 3.1 outcome data available for nearly all participants; 4.1 inappropriate measurement; 4.2 measurement differing between arms; 4.3 assessor awareness; 4.4 assessment could be influenced; 4.5 assessment likely influenced; 5.1 prespecified analysis plan; 5.2 selection from eligible measurements; 5.3 selection from eligible analyses. Responses to conditional questions 2.4, 2.5, 2.7 and 3.2–3.4 were unrecorded (NR). Absence of a recorded response does not establish that a question was inapplicable or that risk of bias was low.

# Supplemental Figures:

Supplemental Figure S1: Risk-of-bias summary for the 19 trial reports representing 18 independent randomised cohorts. D1, bias arising from the randomisation process; D2, bias due to deviations from intended interventions; D3, bias due to missing outcome data; D4, bias in measurement of the outcome; D5, bias in selection of the reported result. This figure presents the study-level risk-of-bias assessments; infection outcome-specific RoB 2 assessments are provided in Supplemental Table S3.

Supplemental Figure S2. Contour-enhanced funnel plot for binary all-cause mortality (14 trials). Points are trial log RRs and standard errors. Dashed limits show ±1.96 standard errors around the inverse-variance centre; shading marks two-sided P contours around no effect (>0.10, 0.05–0.10, 0.01–0.05 and <0.01). Egger t = −0.12, P = 0.903. Zero-event arms use a 0.5 correction for the display and regression. This exploratory test does not exclude selective reporting.

Supplemental Figure S3. Contour-enhanced funnel plot for participants with heart-failure hospitalisation (10 trials). Points are log RRs and standard errors; dashed limits show ±1.96 standard errors around the inverse-variance centre. Shading marks P contours around no effect. Egger t = −1.48, P = 0.176. Zero-event arms use a 0.5 correction for display and regression.

Supplemental Figure S4: Availability of infection outcomes across the 18 independent cohorts. Filled symbols indicate contribution to a pooled analysis; half-filled symbols indicate conditional inclusion; open symbols indicate descriptive data. Missing outcomes were not treated as zero. §Counts reconstructed from rounded percentages. Any-severity and serious-infection patients, episodes, named terms and clinical infection endpoints were classified separately.

Supplemental Figure S5: Quality of life: individual trial effect sizes. Standardised mean differences (Hedges' g) with 95% confidence intervals are shown for seven trials reporting means and standard deviations in both arms. Positive values favour intravenous iron; MLHFQ scores are sign-reversed so that direction is consistent across instruments. No pooled estimate is presented because instruments, score types and assessment times differed and heterogeneity was substantial. EQ-5D, EuroQol 5-Dimension; KCCQ, Kansas City Cardiomyopathy Questionnaire; MLHFQ, Minnesota Living with Heart Failure Questionnaire.

Supplemental Figure S6: Exploratory serious infections by exact MedDRA preferred term. (A) Pneumonia. (B) Sepsis. (C) Urinary tract infection. (D) Bacteraemia. (E) Septic shock. Blue diamonds show binomial-normal GLMM ORs with profile-likelihood CIs; orange diamonds show Mantel–Haenszel ORs. ‡FAIR-HFpEF registry population, N=20/22. Preferred-term patient counts were not summed across diagnoses. *A 0.5 correction was used for trial displays with a zero-event arm; pooled GLMM and Mantel–Haenszel estimates were uncorrected.

Supplemental Figure S7: Leave-one-out infection analyses. (A) Participants with any infection. (B) Any-infection sensitivity analysis including FAIR-HF2. (C) Participants with serious infection. (D) Any-severity infection episodes. (E) Serious infection episodes. The same model was refitted after omitting each trial in turn.

Supplemental Figure S8: Exploratory infection episodes and clinical infection endpoints. (A) Any-severity episodes. (B) Serious episodes, including reporting-window, source and assumed-dispersion sensitivities. (C) IRONMAN trial-reported infection hospitalisation/death estimates and the separate CONFIRM-HF binary hospitalisation-linked TEAE. Episode ratios use participant numbers as exposure and do not represent measured incidence rate ratios. Reporting-window contrasts were descriptive; no interaction test was performed. GLMM confidence intervals use profile likelihood. *A 0.5 correction was used for trial displays with a zero-event arm; pooled GLMM and Mantel–Haenszel estimates were uncorrected. †FAIR-HFpEF any-episode data use publication N=18/21; serious episodes use registry N=20/22. §CONFIRM-HF counts were reconstructed from percentages.

Supplemental Figure S9. Binary clinical outcomes restricted to at least 24 weeks. (A) All-cause mortality, eight trials, including EFFECT-HF and AFFIRM-AHF. (B) HF hospitalisation, seven trials. (C) All-cause hospitalisation, three trials. (D) Cardiovascular hospitalisation, four trials. Orange diamonds show principal common-effect estimates; blue diamonds show REML–mHK sensitivities. SFGC HF readmission counts were reported at six months. A correction is used only for displays and inverse-variance sensitivities when needed; pooled MH estimates are uncorrected.

Supplemental Figure S10. Source and sensitivity analyses for clinical outcomes. (A) All-cause mortality HRs: six-trial analysis, restriction to HRs reported in original trial publications, and leave-one-out analyses. (B) All-cause hospitalisation: principal four-trial analysis, sensitivity analysis including FERRIC-HF event counts and Youssef admissions of unspecified cause, and restriction to follow-up of at least 24 weeks. Toblli 2015 was excluded because the intravenous-iron numerator was unreported. Orange symbols show common-effect estimates; blue symbols show REML–mHK sensitivity estimates.

Supplemental Figure S11. Infection outcomes by trial characteristics. Blue points show GLMM estimates for strata containing at least two trials; grey points represent single trials. Episode outcomes are episode-burden ratios using participants as exposure, not person-time incidence rate ratios. No subgroup-difference tests are reported. IRONMAN included outpatient and hospital recruitment. FAIR-HFpEF registry records are omitted from reporting-window strata because the precise horizon was unconfirmed. Sparse strata and confounding preclude inference about effect modification.

# Supplemental Appendix 1 Search strategies

## Database: Ovid MEDLINE(R) ALL <1946 to April 16, 2026>

Search Strategy:

1	exp Ferric Compounds/ (45644)

2	exp Ferrous Compounds/ (19710)

3	exp Iron/ (118857)

4	(alvofer or colliron or faremio or ferion or feriv or fermed or ferri saccharate or ferric hydroxide sucrose or ferric oxide saccharate or ferric oxide,saccharated or ferric saccharate or ferrinemia or ferrisaccharate or ferrivenin or ferrologic or ferrous saccharate or ferrovin or fesin or hemafer s or hemafer-s or idafer or (iron adj2 hydroxide sucrose complex) or iron saccharate or iron sucrose or ironcrose or iviron or nefro-fer or nefrofer or neo ferrum or nephroferol or proferrin or referen or reoxyl or saccharate ferric or saccharate iron or saccharated ferric oxide or saccharated iron oxide or sucro fer or sucrofer or sucroven or veniron or venofer or venotrix).tw,kf. (1062)

5	(ferric carboxymaltose or Ferinject or Injectafer or Iroprem).tw,kf. (1013)

6	(ferlecit or ferlixit or ferric gluconate or ferrigluconate or ferrlecit or gluconate ferric sodium or (iron adj2 gluconate) or sodium ferrigluconate or intravenous iron sucrose or iron sucrose injection* or venofer).tw,kf. (649)

7	(diafer or ferric derisomaltose or iron isomaltoside or monofer or monafer or monoferro or monover or ferumoxytol or feraheme or rienso).tw,kf. (959)

8	(IV iron or "IV iron" or iron therapy or ((intravenous* or inject* or infus* or parenteral) adj3 iron)).tw,kf. (8186)

9	(ferumoxytol or injectafer or infed or cosmofer or iron polymaltose or triferic or ferric pyrophosphate citrate).mp. (853)

10	(anaemex or cosmofer or dexferrum or dexiron or dextrafer or dextran fe or dextran ferrous or dextran iron or driken or fenate or fer dextran or ferric dextran or ferridex or tranferrisat or ferrodex or ferrodextran or ferrous dextran or ferrum lek or fervetag or hibiron or imferdex or imferon or impheron or imposil or infed or infufer or iron dextran complex or ironate or monofar or proferdex or uniferon or uniferon or uniferron).mp. (1606)

11	1 or 2 or 3 or 4 or 5 or 6 or 7 or 8 or 9 or 10 (170939)

12	exp Administration, Intravenous/ (152179)

13	(intravenous* or IV or "IV" or infus* or inject* or parenteral*).tw,kf. (1930174)

14	12 or 13 (1965704)

15	11 and 14 (19416)

16	exp Heart Failure/ (165035)

17	exp Cardiac Output, Low/ (5652)

18	exp Ventricular Dysfunction/ (46210)

19	exp Ventricular Dysfunction, Left/ (37348)

20	exp Ventricular Dysfunction, Right/ (8022)

21	exp Cardiomyopathies/ (123012)

22	(heart failure or cardiac failure or congestive heart failure or CHF or heart decompensation or cardiac decompensation or cardiac insufficiency or heart insufficiency or ventricular dysfunction or left ventricular dysfunction or right ventricular dysfunction or systolic dysfunction or diastolic dysfunction or HFrEF or HFpEF or HFmrEF or cardiomyopathy or dilated cardiomyopathy or ischemic cardiomyopathy or non-ischemic cardiomyopathy or hypertrophic cardiomyopathy or restrictive cardiomyopathy or cardiac pump failure or myocardial failure or ventricular failure or biventricular failure).tw,kf. (382153)

23	(NYHA class or New York Heart Association or functional class or ejection fraction or LVEF or left ventricular ejection fraction or reduced ejection fraction or preserved ejection fraction or mid-range ejection fraction).tw,kf. (121901)

24	16 or 17 or 18 or 19 or 20 or 21 or 22 or 23 (530848)

25	15 and 24 (1212)

## Database: Ovid Embase <1974 to 2026 Week 15>

Search Strategy:

1	exp Ferric Compounds/ (27206)

2	exp ferrous ion/ (22895)

3	exp Ferrous Compounds/ (22895)

4	exp Iron/ (237208)

5	(alvofer or colliron or faremio or ferion or feriv or fermed or ferri saccharate or ferric hydroxide sucrose or ferric oxide saccharate or ferric oxide,saccharated or ferric saccharate or ferrinemia or ferrisaccharate or ferrivenin or ferrologic or ferrous saccharate or ferrovin or fesin or hemafer s or hemafer-s or idafer or (iron adj2 hydroxide sucrose complex) or iron saccharate or iron sucrose or ironcrose or iviron or nefro-fer or nefrofer or neo ferrum or nephroferol or proferrin or referen or reoxyl or saccharate ferric or saccharate iron or saccharated ferric oxide or saccharated iron oxide or sucro fer or sucrofer or sucroven or veniron or venofer or venotrix).tw,kf. (2514)

6	(ferric carboxymaltose or Ferinject or Injectafer or Iroprem).tw,kf. (2432)

7	(ferric carboxymaltose or Ferinject or Injectafer or Iroprem).mp. [mp=title, abstract, heading word, drug trade name, original title, device manufacturer, drug manufacturer, device trade name, keyword heading word, floating subheading word, candidate term word] (3280)

8	(ferlecit or ferlixit or ferric gluconate or ferrigluconate or ferrlecit or gluconate ferric sodium or (iron adj2 gluconate) or sodium ferrigluconate or intravenous iron sucrose or iron sucrose injection* or venofer).tw,kf. (1846)

9	(ferlecit or ferlixit or ferric gluconate or ferrigluconate or ferrlecit or gluconate ferric sodium or (iron adj2 gluconate) or sodium ferrigluconate or intravenous iron sucrose or iron sucrose injection* or venofer).mp. [mp=title, abstract, heading word, drug trade name, original title, device manufacturer, drug manufacturer, device trade name, keyword heading word, floating subheading word, candidate term word] (2188)

10	(diafer or ferric derisomaltose or iron isomaltoside or monofer or monafer or monoferro or monover or ferumoxytol or feraheme or rienso).tw,kf. (2235)

11	(diafer or ferric derisomaltose or iron isomaltoside or monofer or monafer or monoferro or monover or ferumoxytol or feraheme or rienso).mp. [mp=title, abstract, heading word, drug trade name, original title, device manufacturer, drug manufacturer, device trade name, keyword heading word, floating subheading word, candidate term word] (2961)

12	(IV iron or "IV iron" or iron therapy or ((intravenous* or inject* or infus* or parenteral) adj3 iron)).tw,kf. (13929)

13	(ferumoxytol or injectafer or infed or cosmofer or iron polymaltose or triferic or ferric pyrophosphate citrate).mp. (3172)

14	(anaemex or cosmofer or dexferrum or dexiron or dextrafer or dextran fe or dextran ferrous or dextran iron or driken or fenate or fer dextran or ferric dextran or ferridex or tranferrisat or ferrodex or ferrodextran or ferrous dextran or ferrum lek or fervetag or hibiron or imferdex or imferon or impheron or imposil or infed or infufer or iron dextran complex or ironate or monofar or proferdex or uniferon or uniferon or uniferron).mp. [mp=title, abstract, heading word, drug trade name, original title, device manufacturer, drug manufacturer, device trade name, keyword heading word, floating subheading word, candidate term word] (996)

15	1 or 2 or 3 or 4 or 5 or 6 or 7 or 8 or 9 or 10 or 11 or 12 or 13 or 14 (278421)

16	exp Administration, Intravenous/ (473535)

17	exp intravenous drug administration/ (473535)

18	Administration, Intravenous.mp. [mp=title, abstract, heading word, drug trade name, original title, device manufacturer, drug manufacturer, device trade name, keyword heading word, floating subheading word, candidate term word] (639)

19	(intravenous* or IV or "IV" or infus* or inject* or parenteral*).tw,kf. (2822320)

20	16 or 17 or 18 or 19 (3046214)

21	15 and 20 (27797)

22	exp Heart Failure/ (800198)

23	exp heart left ventricle failure/ (46347)

24	exp heart right ventricle failure/ (21174)

25	exp systolic heart failure/ (28951)

26	exp diastolic heart failure/ (23718)

27	exp acute heart failure/ (34186)

28	exp chronic heart failure/ (800198)

29	exp congestive heart failure/ (152919)

30	exp low cardiac output/ (8003)

31	exp left ventricular dysfunction/ (46347)

32	exp cardiomyopathy/ (289824)

33	exp dilated cardiomyopathy/ (105511)

34	exp hypertrophic cardiomyopathy/ (78296)

35	exp ischemic cardiomyopathy/ (15190)

36	exp restrictive cardiomyopathy/ (30432)

37	(heart failure or cardiac failure or congestive heart failure or CHF or heart decompensation or cardiac decompensation or cardiac insufficiency or heart insufficiency or ventricular dysfunction or left ventricular dysfunction or right ventricular dysfunction or systolic dysfunction or diastolic dysfunction or HFrEF or HFpEF or HFmrEF or cardiomyopathy or dilated cardiomyopathy or ischemic cardiomyopathy or non-ischemic cardiomyopathy or hypertrophic cardiomyopathy or restrictive cardiomyopathy or cardiac pump failure or myocardial failure or ventricular failure or biventricular failure).tw,kf. (644982)

38	(NYHA class or New York Heart Association or functional class or ejection fraction or LVEF or left ventricular ejection fraction or reduced ejection fraction or preserved ejection fraction or mid-range ejection fraction).tw,kf. (253266)

39	22 or 23 or 24 or 25 or 26 or 27 or 28 or 29 or 30 or 31 or 32 or 33 or 34 or 35 or 36 or 37 or 38 (1181847)

40	21 and 39 (2164)

## Scopus Search Strategy:

( TITLE-ABS-KEY ( "ferric compounds" OR "ferrous compounds" OR "ferric ion" OR "ferrous ion" OR "iron compounds" OR alvofer OR colliron OR faremio OR ferion OR feriv OR fermed OR "ferri saccharate" OR "ferric hydroxide sucrose" OR "ferric oxide saccharate" OR "ferric saccharate" OR ferrinemia OR ferrisaccharate OR ferrivenin OR ferrologic OR "ferrous saccharate" OR ferrovin OR fesin OR "hemafer s" OR "hemafer-s" OR idafer OR "iron hydroxide sucrose complex" OR "iron saccharate" OR "iron sucrose" OR ironcrose OR iviron OR "nefro-fer" OR nefrofer OR "neo ferrum" OR nephroferol OR proferrin OR referen OR reoxyl OR "saccharate ferric" OR "saccharate iron" OR "saccharated ferric oxide" OR "saccharated iron oxide" OR "sucro fer" OR sucrofer OR sucroven OR veniron OR venofer OR venotrix OR "ferric carboxymaltose" OR ferinject OR injectafer OR iroprem OR ferlecit OR ferlixit OR "ferric gluconate" OR ferrigluconate OR ferrlecit OR "gluconate ferric sodium" OR "iron gluconate" OR "sodium ferrigluconate" OR "intravenous iron sucrose" OR "iron sucrose injection" OR "iron sucrose injections" OR diafer OR "ferric derisomaltose" OR "iron isomaltoside" OR monofer OR monafer OR monoferro OR monover OR ferumoxytol OR feraheme OR rienso OR "IV iron" OR "iron therapy" OR "intravenous iron" OR "parenteral iron" OR "injected iron" OR "infused iron" OR infed OR cosmofer OR "iron polymaltose" OR triferic OR "ferric pyrophosphate citrate" OR anaemex OR dexferrum OR dexiron OR dextrafer OR "dextran fe" OR "dextran ferrous" OR "dextran iron" OR driken OR fenate OR "fer dextran" OR "ferric dextran" OR ferridex OR tranferrisat OR ferrodex OR ferrodextran OR "ferrous dextran" OR "ferrum lek" OR fervetag OR hibiron OR imferdex OR imferon OR impheron OR imposil OR infufer OR "iron dextran complex" OR ironate OR monofar OR proferdex OR uniferon OR uniferron ) ) AND ( TITLE-ABS-KEY ( "intravenous administration" OR "intravenous drug administration" OR "parenteral administration" OR intravenous* OR infus* OR inject* OR parenteral* ) ) AND ( TITLE-ABS-KEY ( "heart failure" OR "cardiac failure" OR "congestive heart failure" OR CHF OR "heart decompensation" OR "cardiac decompensation" OR "cardiac insufficiency" OR "heart insufficiency" OR "ventricular dysfunction" OR "left ventricular dysfunction" OR "right ventricular dysfunction" OR "systolic dysfunction" OR "diastolic dysfunction" OR HFrEF OR HFpEF OR HFmrEF OR "heart failure with reduced ejection fraction" OR "heart failure with preserved ejection fraction" OR "heart failure with mid-range ejection fraction" OR cardiomyopathy OR cardiomyopathies OR "dilated cardiomyopathy" OR "ischemic cardiomyopathy" OR "ischaemic cardiomyopathy" OR "non-ischemic cardiomyopathy" OR "non-ischaemic cardiomyopathy" OR "hypertrophic cardiomyopathy" OR "restrictive cardiomyopathy" OR "cardiac pump failure" OR "myocardial failure" OR "ventricular failure" OR "biventricular failure" OR "low cardiac output" OR "acute heart failure" OR "chronic heart failure" OR "NYHA class" OR "New York Heart Association" OR "ejection fraction" OR LVEF OR "left ventricular ejection fraction" OR "reduced ejection fraction" OR "preserved ejection fraction" OR "mid-range ejection fraction" ) )

## Trial registry searches

Registry searches identified additional completed and ongoing trials. Condition and intervention fields were combined with AND. No infection-outcome filter was applied. Registry records and linked primary-register records were examined for adverse-event results through 18 October 2026.

## ClinicalTrials.gov

Platform: https://clinicaltrials.gov/ (Advanced Search).

Condition or disease field: "heart failure" OR "cardiac failure" OR "congestive heart failure" OR HFrEF OR HFmrEF OR HFpEF OR "left ventricular dysfunction" OR "ventricular systolic dysfunction"

Intervention or treatment field: iron OR "intravenous iron" OR "parenteral iron" OR "iron infusion" OR "ferric carboxymaltose" OR "ferric derisomaltose" OR "iron isomaltoside" OR "iron isomaltoside 1000" OR "iron sucrose" OR "ferric gluconate" OR "sodium ferric gluconate" OR ferumoxytol OR "iron dextran" OR "iron polymaltose" OR Ferinject OR Injectafer OR Monofer OR Monoferric OR Venofer OR Feraheme

Limits: interventional studies; all recruitment statuses; no restriction by results availability, phase, country, age or sex. Registration dates were unrestricted up to the search date.

## WHO International Clinical Trials Registry Platform

Platform: https://trialsearch.who.int/ (Advanced Search).

Condition field: ("heart failure" OR "cardiac failure" OR "congestive heart failure" OR HFrEF OR HFmrEF OR HFpEF OR "left ventricular dysfunction" OR "ventricular systolic dysfunction")

Intervention field: (iron OR "intravenous iron" OR "parenteral iron" OR "iron infusion" OR "ferric carboxymaltose" OR "ferric derisomaltose" OR "iron isomaltoside" OR "iron isomaltoside 1000" OR "iron sucrose" OR "ferric gluconate" OR "sodium ferric gluconate" OR ferumoxytol OR "iron dextran" OR "iron polymaltose" OR Ferinject OR Injectafer OR Monofer OR Monoferric OR Venofer OR Feraheme)

Limits: all recruitment statuses; no restriction by phase, results availability or country. Registration dates were unrestricted up to the search date.

## Retrieval of registry safety results

Records were cross-linked by trial acronym, title and registration identifier. Linked ClinicalTrials.gov and EU Clinical Trials Register results and associated public documents were examined for broad infection categories, individual infection terms, serious and non-serious adverse events, affected participants, event counts, safety denominators and reporting windows. WHO ICTRP records were used to locate the corresponding primary-register records and linked results. Duplicate reports of the same trial were reconciled rather than counted as independent data. Missing or unlisted infection outcomes were not treated as zero. Source-specific extraction locations and reporting limitations are documented in Supplemental Table S6.

## Supplementary searches of regulatory sources

The EMA and FDA websites, including Drugs@FDA, were searched as part of supplementary safety-source retrieval through 18 October 2026. Searches used generic and proprietary names of relevant intravenous iron preparations, supplemented by trial acronyms, registration identifiers and sponsor study identifiers where available. The EMA search included public assessment and referral material; the FDA search included publicly available approval and review documents. These were supplementary searches for trial-level safety information, not searches of spontaneous-reporting databases.

| **Preparation** | **Generic and proprietary search concepts** |
| --- | --- |
| Ferric carboxymaltose | ferric carboxymaltose; Ferinject; Injectafer |
| Ferric derisomaltose | ferric derisomaltose; iron isomaltoside; iron isomaltoside 1000; Monofer; Monoferric |
| Iron sucrose | iron sucrose; Venofer |
| Sodium ferric gluconate | sodium ferric gluconate; ferric gluconate; Ferrlecit |

## Detailed EMA and FDA search terms

A contemporaneous record of the exact regulatory queries was unavailable. The strategy below was reconstructed from documented product terms and retrieval methods, with trial identifiers cross-referenced to Supplemental Table S6. The regulatory search cutoff was 18 October 2026.

EMA: website search at https://www.ema.europa.eu/en/medicines, including relevant public assessment and referral material. Separate product-name entries: “ferric carboxymaltose”; “Ferinject”; “Injectafer”; “ferric derisomaltose”; “iron isomaltoside”; “iron isomaltoside 1000”; “Monofer”; “Monoferric”; “iron sucrose”; “Venofer”; “sodium ferric gluconate”; “ferric gluconate”; “Ferrlecit”.

FDA: FDA website and Drugs@FDA at https://www.accessdata.fda.gov/scripts/cder/daf/, including public approval and review documents. Separate product-name entries: “ferric carboxymaltose”; “Injectafer”; “ferric derisomaltose”; “Monoferric”; “iron isomaltoside”; “iron isomaltoside 1000”; “iron sucrose”; “Venofer”; “sodium ferric gluconate”; “ferric gluconate”; “Ferrlecit”. Product entries were not required to contain an infection term.

Trial and study identifiers for locating and checking relevant records: “FAIR-HF”; “CONFIRM-HF”; “EFFECT-HF”; “AFFIRM-AHF”; “IRONMAN”; “HEART-FID”; “FAIR-HFpEF”; “FAIR-HF2”; “FER-CARS-05”; “FER-CARS-03”; “FER-CARS-01”. Registration identifiers documented in Supplemental Table S6 include NCT02937454, NCT03037931, 2011-001695-19, 2011-000603-40 and 2015-005757-12. These identifiers link source records; they are not additional independent trials.

Within-document search terms: “heart failure”; “infection”; “infections”; “infections and infestations”; “adverse events”; “serious adverse events”; “safety”, together with the relevant trial acronym or study identifier. Terms separated by semicolons denote individual search entries or document lookups, not a single Boolean query. Product searches and subsequent document searches were separate stages.

Regulatory access points: EMA medicines and referral information, https://www.ema.europa.eu/en/medicines; FDA Drugs@FDA, https://www.accessdata.fda.gov/scripts/cder/daf/. Supplementary regulatory retrieval cutoff: 18 October 2026.
